# Methylation-Based ctDNA Tumor Fraction Changes Predict Long-Term Clinical Benefit From Immune Checkpoint Inhibitors in RADIOHEAD, a Real-World Pan-Cancer Study

**DOI:** 10.1158/2767-9764.CRC-25-0151

**Published:** 2025-08-20

**Authors:** Samantha I. Liang, Zoe Quandt, Sara Wienke, Jing Wang, Sean Gordon, Reagan M. Barnett, Jude Masannat, Kyle Chang, Carin R. Espenschied, Katie J. Quinn, Kimberly C. Banks, Enjun Yang, John E. Connolly

**Affiliations:** 1 Parker Institute for Cancer Immunotherapy, San Francisco, California.; 2 Division of Endocrinology and Metabolism, Department of Medicine, UCSF, San Francisco, California.; 3 Guardant Health, Palo Alto, California.

## Abstract

Current response evaluation methods have accuracy limitations. Monitoring with a ctDNA methylation-based tumor fraction (TF) may provide a more accurate assessment of tumor burden across solid tumors. In this study, we evaluate a tissue-free, methylation-based TF and explore the association with treatment outcomes in RADIOHEAD, a cohort of 1,070 patients with solid tumors receiving standard-of-care immune checkpoint inhibition (ICI) regimens, with blood samples collected prospectively for retrospective analysis. A total of 1,997 baseline and serial on-treatment plasma samples from 627 patients with stage IV cancer were analyzed with an analytically validated next-generation sequencing methylation-based ctDNA assay (Guardant Reveal). The primary outcome measure was real-world progression-free survival (rwPFS). Secondary outcomes included real-world overall survival (rwOS) and the lead time between nonmolecular response (nMR) to rwPFS event. Patients with any decrease in TF while receiving ICI had superior outcomes. Patients with ≥80% decrease in TF at two timepoints or TF below the limit of quantification had longer rwPFS and rwOS than those with <80% decrease [nMR; rwPFS HR, 0.24 (95% confidence interval, 0.19–0.32) *P* < 0.005; rwOS HR, 0.28 (95% confidence interval, 0.21–0.38) *P* < 0.005]. nMR was detected prior to clinical progression in 209 patients with a median lead time of 3.03 months. Among 627 patients with stage IV cancer receiving standard-of-care ICI, monitoring with methylation-based TF identified patients who have significantly longer rwPFS and rwOS. Changes in TF on ICI can provide additional response data earlier than imaging alone and support the potential of serial monitoring to inform treatment decisions.

**Significance:**

This study found that early decreases in tumor DNA levels in the blood are linked to longer survival in patients with cancer treated with immunotherapy. These findings support the use of blood-based monitoring to help predict treatment response and improve decision-making in real-world cancer care.

## Introduction

Real-world studies are important for the evaluation of biomarkers that guide treatment choices, particularly in community oncology settings in which blood-based assays would be a practical alternative to tissue-based tests (1, 2). The **R**esistance **D**rivers for **I**mmuno-**O**ncology Patients Interrogated by **H**armonized Mol**e**cul**a**r **D**atasets (RADIOHEAD) study exemplifies this approach, enrolling 1,070 patients across community oncology clinics and collecting more than 3,500 blood samples longitudinally for biomarker evaluation (3). ctDNA liquid biopsy provides a minimally invasive approach to identify genomic determinants of treatment response and resistance at both diagnosis and progression without requiring tissue, thus improving patient access to targeted therapies (4). In addition, plasma ctDNA evaluation has a faster result delivery, in which the typical time between shipment and results is a median of 7 to 9 days, 6 to 12 days sooner than that for tissue-based tests (5, 6), ensuring timely administration of optimal therapies (1, 7) and consequently improved patient outcomes (8). Data from the SCRUM-Japan GOZILA project, initiated in 2018, demonstrated that up to 24% of patients with gastrointestinal tumors received targeted therapy based on liquid biopsy results, leading to a survival rate approximately twice that of patients who did not receive liquid biopsy–guided treatment (9). Liquid biopsy is now standard-of-care (SoC) for biomarker detection, and multiple liquid biopsy investigations demonstrated associations between treatment-induced changes in ctDNA levels and overall survival (OS) and progression detection earlier than standard approaches (10–15).

Establishing responsiveness to therapy and detecting progression is vital to treatment decisions for patients with advanced cancer. In common solid tumor malignancies, radiologic imaging is currently used, and when available, blood proteins. The most widely utilized and validated response criteria is the RECIST, which measures changes in tumor burden based on radiologic imaging (16). iRECIST was later introduced to address the challenges with pseudoprogression when evaluating response to immunotherapy (17). RECIST has limitations, including inter-reader reliability of radiologic images, requiring measurable disease at baseline, constraints regarding selection of target lesions, detecting new lesions, inability to accurately measure bone metastases and body cavity–based disease such as pleural effusion and ascites, and for patients with stable disease (SD), uncertainty about clinical benefit (18). Therefore, improved methods of clinical response evaluation are needed.

Liquid biopsy assays have been studied as a method for evaluating molecular response (MR) to therapy (12, 13, 19–29), and ctDNA levels may be easily monitored with simple blood draws throughout a patient’s journey to provide early information on response and progression. Liquid biopsy has also been explored as a tool to distinguish between pseudoprogression and true progression in patients receiving immune checkpoint inhibitors (ICI), complementing imaging-based response assessments (13, 30–32). Furthermore, patients deemed to have SD by RECIST may be further adjudicated by ctDNA results (12). Liquid biopsy assays have thus far focused on genomic analysis, using variant allele fraction changes for monitoring. This approach has limitations, including reduced sensitivity at low ctDNA levels and potential interference from copy-number variation and clonal hematopoiesis (26, 29, 33). Tumor-informed ctDNA monitoring assays have been developed to address these limitations, but this approach requires sufficient tissue, which may affect evaluability and increase turnaround time. Methylation-based ctDNA quantification has been explored as an alternative approach. This study evaluates a tissue-free, methylation-based next-generation sequencing assay for quantifying ctDNA levels and assessing correlations between changes in tumor fraction (TF) and patient outcomes in the real-world pan-cancer RADIOHEAD study.

## Materials and Methods

### Patients and study design

RADIOHEAD is a cohort study of 1,070 patients with cancer receiving SoC ICI regimens, with longitudinal blood samples collected prospectively for retrospective analysis. Briefly, subjects with solid tumors naïve to ICIs were enrolled from 49 US oncology clinics from May 2018 to May 2022. Blood and clinical data were prospectively collected at baseline, immediately prior to cycle 3, and at 6 and 12 months, with additional collections at onset of immune-related adverse event (irAE) and follow-up if applicable (time of irAE incidence, 4–6 weeks after irAE, and 6 and 12 months after irAE). Exact blood collection timing varied based on clinical care, and length of treatment cycle was defined by clinical SoC. Clinical data, including demographics, medical history, cancer stage at the time of study enrollment, treatment details, and irAE information, were recorded and managed in a centralized REDCap (34) database by research site coordinators at each site (3). Clinical data were reviewed for accuracy and mapped to standard ontologies, and study timepoints were standardized relative to therapy start. A multistage quality control (QC) process excluded patients without confirmed immunotherapy-naïve status or with fewer than 30 days on study. For survival analyses, real-world OS (rwOS) was calculated from the start of ICI treatment to either the date of death or last contact, with data truncated at 15 months to minimize bias from extended follow-up of certain patients. For progression analyses, a real-world progression-free survival (rwPFS) variable was derived using documented progression, death, treatment change, or censoring at last contact dates. Death and progression dates were recorded verbatim, with imputation only in specific cases—if a patient’s off-study reason was listed as “death” without a recorded date (imputed as the off-study date) or “transition to hospice” (imputed as the progression date). Probable progression events were identified through treatment change forms indicating therapy escalations, such as adding ipilimumab to PD-1 monotherapy, switching from ICIs to chemotherapy, or starting alternative therapies (i.e., tyrosine kinase inhibitors). Treatment de-escalations, such as moving from combination ICI therapy to monotherapy, were classified as unlikely progression. For rwPFS, the treatment change date was used if it preceded other clinical observation dates; otherwise, the earliest clinical observation date was selected. Effective off-study events were censored unless clinic notes indicated progression, and death or hospice transitions were counted as progression events unless an earlier progression date was recorded. Investigators obtained written informed consent from each participant. The study was conducted in accordance with recognized ethical guidelines, and Institutional Review Board approval was obtained through WCG Institutional Review Board Pr. No. 20182579.

**Figure 1:**
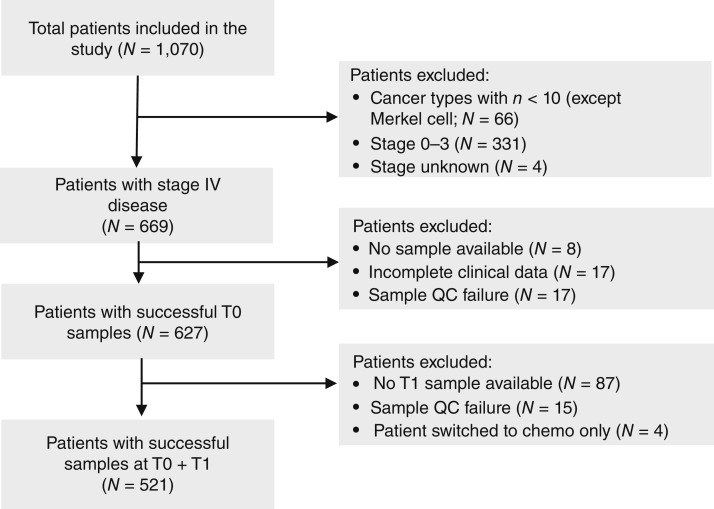
Strobe diagram showing inclusion and exclusion for patient samples from the RADIOHEAD study in which successful samples passed all QC metrics and delivered a TF result. Chemo, chemotherapy.

From the overall cohort, 627 patients with stage IV non-small cell lung cancer (NSCLC), small cell lung cancer (SCLC), renal cell carcinoma (RCC), malignant melanoma, bladder cancer, head and neck squamous cell carcinoma, gastroesophageal carcinoma, hepatocellular carcinoma, endometrial carcinoma, colorectal carcinoma, prostate cancer, breast cancer, and Merkel cell carcinoma with complete clinical data and baseline plasma sample available, passing all sequencing QC metrics, yielding a TF value ≥ 0, were eligible for evaluation (Fig. 1). ICI regimens received are summarized by cancer type in Supplementary Table S1. Patients with stages 0 to III cancer were excluded because of the limited nature of prestudy clinical data and the inability to confirm which patients were receiving adjuvant ICI versus those receiving palliative ICI.

### Plasma sample analysis and liquid biopsy next-generation sequencing assay

For this study, cell-free DNA (cfDNA) was extracted from 1mL of plasma which was collected in EDTA tubes and processed at a centralized site. There, the samples were stored frozen until delivery to Guardant Health for analysis. cfDNA methylation analysis was performed using Guardant Reveal powered by the Guardant Infinity platform (Guardant Health). Briefly, up to 30 ng of cfDNA was tagged with molecular identifiers, physically partitioned based on methylation status using bead-based methylation binding domain affinity, and clarified using methylation-specific restriction endonuclease treatment. Samples were then enriched for CpG-dense regulatory regions unmethylated in cfDNA derived from healthy patients and sequenced on the Illumina platform. ctDNA detection and quantification used a 15 MB panel comprising more than 20,000 differentially methylated regions. Per region methylation signal was normalized relative to internal control regions. Detection threshold selection was performed for each tumor region set using a training set of more than 5,000 cancer-free donor samples, ctDNA-free samples, and samples from individuals with cancer. Sequencing QC metrics include read quality, total yield, coverage, and mapping rate. For those samples passing QC metrics, TF was defined by comparison of observed methylation signals to methylation levels across constitutively methylated and unmethylated regions within each sample.

### MR threshold

The optimal ctDNA change threshold distinguishing MR from non-MR (nMR) was determined using a predefined training set. A power analysis was conducted using the *lifelines* library’s sample size calculation function for Cox proportional hazards (CPH) models. We modeled a HR of 2 between TF responders and nonresponders as a conservative estimate, informed by earlier studies. Under 80% and 90% power targets (at a conventional α of 0.05), total sample sizes of 178 and 238 participants, respectively, were predicted to be sufficient. In a secondary scenario, using a higher HR of 3 and a fixed group size of 50 per arm (responder/nonresponder), we observed an approximate 88% power. For the MR threshold determination, participants were assigned to training and validation subsets in a 60:40 ratio, using a data-splitting approach that stratified individuals by the occurrence of a rwPFS event, ensuring balanced representation of progressions across the two subsets. The final training and test participant sizes were 382 and 245, respectively. Thus, both the training and test cohorts would provide adequate power to detect HRs of two or greater according to the predetermined analyses in sample size determination. Both training and test groups were balanced for clinical characteristics (Table 1).

**Table 1:** Clinical characteristics of the patients in the evaluable cohort

Characteristic	Total cohort	Training set	Test set
Stage IV patients with successful samples *n* (%)	​	​	​
Baseline	627 (100)	382 (100)	245 (100)
T1 (15–105 days)	521 (83)	312 (82)	209 (85)
T2 (106–272 days)	335 (53)	188 (49)	147 (0.6)
T3 (273–450 days)	226 (36)	129 (34)	97 (40)
Age (years)	​	​	​
Mean	68.8	68.6	69.0
Median (range)	69 (29–89)	69 (29–89)	69 (32–89)
Gender *n* (%)	​	​	​
Male	376 (60)	226 (59)	150 (61)
Female	251 (40)	156 (41)	95 (39)
Race *n* (%)	​	​	​
White	568 (91)	347 (91)	221 (90)
Black	36 (6)	22 (6)	14 (6)
Asian	14 (2)	8 (2)	6 (2)
Native American	3 (0.5)	1 (0.3)	2 (0.8)
Pacific Islander	1 (0.2)	1 (0.3)	0 (0)
Other	5 (0.8)	3 (0.8)	2 (0.8)
Tobacco use *n* (%)	​	​	​
Never	162 (26)	93 (24)	69 (28)
Former	358 (57)	221 (58)	137 (56)
Current	107 (17)	68 (18)	39 (16)
On-study treatment	​	​	​
Immunotherapy	434 (69)	260 (68)	174 (71)
Immunotherapy + chemotherapy	177 (28)	112 (29)	65 (27)
Immunotherapy + other	16 (2.5)	10 (2.6)	6 (2.4)
Any irAE	​	​	​
Yes	149 (24)	86 (23)	63 (26)
No	478 (76)	296 (77)	182 (74)
Best overall RECIST outcome at 6-month visit	​	​	​
Not provided	542 (86)	331 (87)	211 (86)
CR	6 (1)	2 (0.5)	4 (1.6)
PR	16 (3)	9 (2.4)	7 (3)
SD	37 (6)	26 (7)	11 (5)
PD	26 (4)	14 (4)	12 (5)

For threshold determination, a subset of patients with successful baseline (T0) and early on-treatment samples (T1), in which at least one of those was above the limit of quantification (LOQ) was used. This resulted in an analyzable subset of 459 patients, comprising 273 from the training set and 186 from the test set. These participants were drawn from the same original cohorts, with exclusions applied solely based on the sample success and LOQ criterion for this specific analysis. The optimal threshold was determined by a balance of selecting the lowest HR, shortest rwPFS for the nMR group, as well as the largest number of patients identified with MR across a range of thresholds from 0% to 100%. Threshold definitions for TF changes were finalized using only the training set, and those predefined thresholds were then applied to the test set. This approach enabled data-driven threshold selection in the training partition while preventing overfitting in the test partition and full cohort analysis.

Subsequent longitudinal monitoring analyses were performed on the combined set of all evaluable patients. For the purpose of analysis, all blood samples collected 15 days or later on treatment were included and grouped into on-treatment timepoints [timepoint 1 (T1: 15–105 days), timepoint 2 (T2: 106–272 days), and timepoint 3 (T3: 273–450 days)]. These groupings were defined empirically to maximize the number of evaluable patients while aligning with typical clinical follow-up intervals. A sensitivity analysis was conducted using narrower time ranges to assess whether alternate grouping strategies affected downstream analyses. Subgroup analyses examined differences in response prediction for patients receiving ICI alone versus ICI plus chemotherapy (T1 only) and across individual cancer types (T1, T2, or T3) to ensure consistent threshold performance. For comparisons between two timepoints, when multiple timepoints were available within the range, the earliest sample within that timeframe was used for analysis.

### Longitudinal monitoring analysis

Patients with both successful T0 and T1 samples that remained on ICI therapy for the duration were included (*N* = 521), regardless of ctDNA detection at T0, to evaluate changes in TF over time and their association with outcomes. MR was defined by the threshold (decrease in TF) determined above at either T1, T2, or T3 from the prior timepoint. nMR was defined as a TF decrease less than that threshold. TF values below the LOQ (0.01%) at both timepoints were combined with the respective relative decrease group unless otherwise specified. For comparisons in which a single timepoint was below the LOQ, the TF value of 0.01% was used for change calculations.

For a subset of patients (*n* = 75), clinician-reported “overall RECIST best outcome” was available at their 6-month visit. Although direct imaging data and scan dates were not collected, this allowed for comparison with MR based on TF. For the best MR analysis, the greatest TF decrease was calculated for each sample available within 15 to 272 days of treatment, selecting the lowest value (greatest decrease) to generate a waterfall plot. The best MR was compared with RECIST outcome using a *χ*^2^ test. Changes in ctDNA were calculated as follows:

$$\left(\frac{TF}{prior\;TF}-1\right)\;\times 100$$

### Lead time analysis

Lead time for nMR was calculated as the time from the first sign of nMR (after initial MR, if applicable) to rwPFS event. For patients with nMR and a sample available only at T1, lead time was calculated between T1 and rwPFS event. For those with nMR at both T1 and T2, lead time was calculated from T1 to rwPFS event. For those that had MR at T1 and nMR at T2, lead time was calculated from T2 to rwPFS event. For patients with all three timepoints, if MR was observed at T1 and T2, then lead time was calculated from T3 to rwPFS event. For those that had nMR at T1 and T2, lead time was calculated from T1 to rwPFS event. For those that had MR at T1 and nMR at T2, lead time was calculated from T2 to rwPFS event. Lastly, for patients with nMR at T1 and MR at T2, lead time was calculated from T3 to rwPFS event. Patients with missing baseline or intervening timepoints or no rwPFS event were excluded from this analysis.

### Statistical analysis

Analysis was conducted according to a predefined data analysis plan. The primary outcome measure was rwPFS, with secondary outcome measures rwOS and lead time between nMR and real-world progression event.

Descriptive statistics summarized patients and clinical characteristics, reporting median and range for continuous variables, and frequencies and percentages for categorical variables. Median rwPFS and rwOS were calculated in months for both MR and nMR groups and analyzed with the Kaplan–Meier method. Because on-treatment samples were drawn at different times relative to progression, rwPFS and rwOS were reindexed to start from the on-treatment blood draw to account for potential survival bias.

A CPH model was generated and included gender, age, cancer type (NSCLC vs. non-NSCLC), irAE, current tobacco use, prior treatment (chemotherapy, targeted therapy, and/or radiation before enrollment), and baseline TF (continuous variable). HRs and *P* values were adjusted to account for potential confounder covariates, and the concordance index was calculated to assess the model’s accuracy in differentiating survival outcomes. The results were considered statistically significant if the adjusted *P* value (*P*) was ≤ 0.05, and a concordance index value of >0.7 was considered indicative of a good model.

Baseline TF detection, quantification, and distribution figures were generated using GraphPad Prism version 10.4.1 for macOS, GraphPad Software. CPH and Kaplan–Meier statistical modeling were performed with the *lifelines* package version 0.27.7. General statistical analyses and figures were generated using Python version 3.7.6 and R version 4.2.1.

### Data availability

Raw sequencing data for this study were generated at Guardant Health and are not publicly available due to their proprietary nature and patient privacy concerns. De-identified patient-level clinical and methylation data required to recapitulate study results are available in Supplementary Table S2, and additional details may be available through the corresponding author upon reasonable request.

## Results

### Cohort overview

Among 669 patients with stage IV disease, 644 with complete clinical data and available samples contributed to the analyzed sample set, *N* = 1,761, including NSCLC (*N* = 230, 36%), RCC (*N* = 81, 13%), malignant melanoma (*N* = 60, 9%), SCLC (*N* = 56, 9%), bladder cancer (*N* = 47, 7%), head and neck squamous cell carcinoma (*N* = 46, 7%), or other solid tumors (*N* = 124, 19%). The median patient age was 69 years, 60% were male, most patients were White (91%), and 69% received ICI alone (Table 1). The distribution of sample collection timing is shown in Supplementary Fig. S1. Samples were grouped into T1 = 15 to 105 days, T2 = 106 to 272 days, and T3 = 273 to 450 days for TF change analyses by using the empirical distribution. The number of patients with successful samples at each timepoint, categorized by cancer type, is summarized in Supplementary Table S3. Notably, 644 patients had complete clinical data and a baseline plasma sample, with 97.4% (627/644) of those samples successfully processed using the Guardant Reveal assay on the Guardant Infinity platform, yielding a TF result. In total, 97% (1,709/1,761) samples processed across timepoints were successful. All patients in the study were receiving their first line of ICI treatment, though some had prior therapies. Information on prior treatments, on-study regimens, and the timing of on-treatment samples relative to baseline, rwPFS, and rwOS events are illustrated in swimmer plots (Supplementary Fig. S2).

### Baseline TF is prognostic

ctDNA was detected in 86% (542/627) of successful baseline (pretreatment) plasma samples, with TF quantifiable in 85% (532/627) across various cancer types using methylation-based detection methods. Detection rates were highest in hepatocellular carcinoma (100%), prostate cancer (100%), Merkel cell carcinoma (100%), and SCLC (98%). The lowest detection rates were observed in RCC (64%), malignant melanoma (81%), and NSCLC (86%; Supplementary Fig. S3A). The median TF varied by cancer type, with the highest values observed in SCLC (21.05%), breast cancer (16.85%), prostate cancer (11.71%), and bladder cancer (4.06%). The lowest median TF values were seen in RCC (0.39%), gastroesophageal cancer (0.66%), and NSCLC (0.86%; Supplementary Fig. S3B; Supplementary Table S4).

Patients with ctDNA below the LOQ at baseline had significantly better outcomes, with rwPFS and rwOS not reached (NR) compared with patients in the highest baseline percentile [median rwPFS 5.30 months; HR, 0.29 (95% confidence interval (CI), 0.192–0.434) log-rank *P* < 0.005; median rwOS 7.30 months; HR, 0.23 (95% CI, 0.136–0.373) log-rank *P* < 0.005; Supplementary Fig. S3C and S3D]. Although baseline TF was a strong prognostic factor, changes in TF from baseline were significantly associated with rwPFS across all levels of baseline TF (Supplementary Fig. S3E–S3H).

### MR threshold analyses

**Figure 2:**
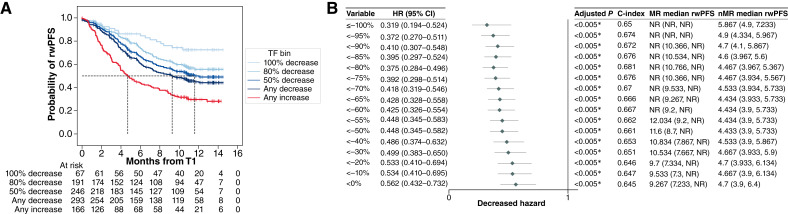
**A** and **B,** Candidate thresholds for TF change indicating MR and nMR among the patients in whom either T0 or T1 were quantifiable (*n* = 459). **A,** Forest plot for various TF changes (**B**) Kaplan–Meier analysis of rwPFS by select candidate thresholds across the full dataset. Log-rank test between groups: 100% vs. any decrease, *P* < 0.005; 80% decrease vs. any decrease, *P* = 0.01; 100% decrease vs. 80% decrease, *P* = 0.04; 100% decrease vs. any increase, *P* < 0.005; 80% decrease vs. any increase, *P* < 0.005; any decrease vs any increase, *P* < 0.005. c-index, concordance index.

To determine what TF reduction best stratifies MR from nMR, we assessed incremental thresholds from 0% to 100%. The optimal MR threshold for prediction of outcomes was determined in the training set and confirmed in the test set (Supplementary Fig. S4A and S4B). Patients with any level of ctDNA decrease during ICI treatment had a statistically significant improvement in PFS (*P* < 0.005; Fig 2A and B). At T1, patients with MR (*n* = 252) had significantly longer median rwPFS compared with those without MR [*n* = 268; NR (95% CI, NR–NR) vs. 4.47 months (95% CI, 3.97–5.37); adjusted HR 0.35 (95% CI, 0.27–0.45) *P* < 0.005]. Similarly, MR was associated with significantly improved rwOS compared with nMR [NR (95% CI, NR–NR) vs. 10.27 months (95% CI, 8.40–11.90); adjusted HR 0.39 (95% CI, 0.29–0.54) *P* < 0.005]. Patients with 100% decrease in TF had better outcomes compared with those with ≥80% decrease (*P* = 0.04; Fig. 2A and B). However, the median rwPFS of the nMR group was 4.5 months with ≥80% decrease, whereas the median rwPFS of the nMR group was longer at 5.9 months with 100% decrease. Additionally, ≥80% decrease identified three times more patients with MR than 100% decrease. Therefore, ≥80% decrease was identified as the optimal threshold for stratifying MR from nMR. This trend was confirmed in the reserved test set in which the ≥80% threshold was statistically significant (adjusted *P* = 0.03; Supplementary Fig. S4B). These associations were consistent across treatment groups, regardless of whether the patients received ICI alone or in combination with chemotherapy (Supplementary Table S5). Patients with TF below LOQ at both timepoints showed similar outcomes as those with ≥80% decrease and were therefore included in the MR group for subsequent analyses (Supplementary Fig. S5). Additionally, patients with <80% decrease and those with TF increase had very similar outcomes, further supporting the 80% threshold as the optimal threshold for cohort stratification (Supplementary Fig. S5).

### Longitudinal assessment of TF predicts ICI benefit in a pan-solid tumor cohort

**Figure 3:**
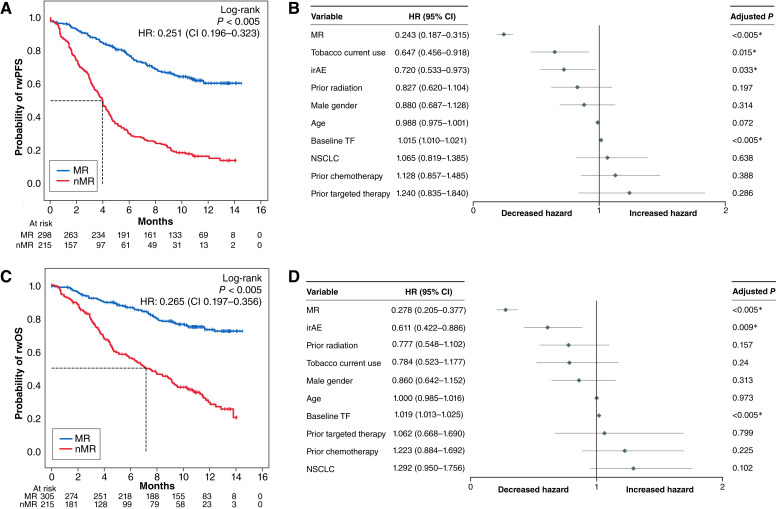
**A–D,** Longitudinal assessment of TF predicts ICI benefit in a pan-solid tumor cohort (*n* = 521; patients in whom rwPFS or rwOS event occurred before the reindex date were excluded where indicated). **A,** Kaplan–Meier analysis comparing the rwPFS of MR at any timepoint [NR (95% CI, NR–NR)] vs. nMR at all timepoints [3.93 months (95% CI, 3.33–4.40); *n* = 513]. **B,** Forest plot for CPH model for rwPFS with a HR of 0.24 (0.19–0.315) for MR vs. nMR (*n* = 513). **C,** Kaplan–Meier analysis comparing the rwOS of MR at any timepoint vs. nMR at all timepoints [NR (95% CI, NR–NR) vs. 7.166 months (95% CI, 5.73–9.10; *n* = 520)]. **D,** Forest plot for CPH model for rwOS with a HR of 0.28 (0.21, 0.38) for MR vs. nMR (*n* = 520).

A total of 521 patients were included in this longitudinal analysis. Patients with MR at any timepoint had significantly longer rwPFS and rwOS compared with those without MR (nMR) at all timepoints. The median rwPFS was NR for the MR group (95% CI, NR–NR) versus 3.93 months for the nMR group (95% CI, 3.33–4.40), with an adjusted HR of 0.24 (95% CI, 0.18–0.32; adjusted *P* < 0.005; Fig. 3A and B). Similarly, the median rwOS was NR (95% CI, NR–NR) versus 7.17 months (95% CI, 5.73–9.10), with an adjusted HR of 0.27 (95% CI, 0.21–0.38; adjusted *P* < 0.005; Fig. 3C and D). A sensitivity analysis using alternate time ranges showed similar results but limited the evaluable patient population (Supplementary Table S6). In multivariate analysis, after adjusting for baseline TF, changes in TF remained strongly associated with both rwPFS and rwOS (Fig. 3B and D).

Evaluating MR across all on-treatment timepoints identified 21% (53/252) more patients achieving MR compared with assessments based only on the first on-treatment sample (T1). The association between TF changes and outcome was observed across cancer types evaluated (Supplementary Table S7).

Change in TF preceded rwPFS events, with a median lead time from nMR to rwPFS of 3.03 months. Among 214 patients, nMR was detected before rwPFS events in 209 cases (97.7%). The median lead time varied by when nMR was first detected: 2.47 months for those with nMR at T1 (*n* = 124), 4.20 months for those detected at T2 (*n* = 69), and 4.67 months for those detected at T3 (*n* = 16).

### TF change on ICI complements RECIST best overall response at 6 months

**Figure 4:**
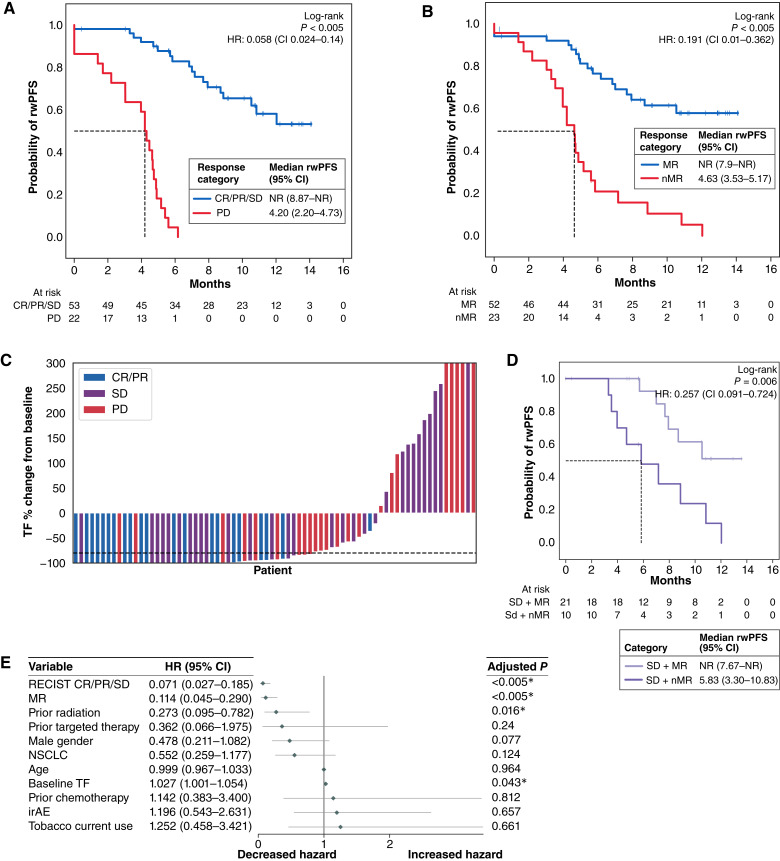
**A–E,** MR outcome prediction is complementary to RECIST in subset of patients with 6-month best RECIST response available (*n* = 75). Kaplan–Meier plots for rwPFS. **A,** RECIST association with rwPFS by two groups combining CR/PR/SD vs. PD. **B,** MR and nMR at any timepoint association with rwPFS in the RECIST subset. **C,** Waterfall plot of greatest percentage TF change (best MR) within 6 months by RECIST response. **D,** SD patients stratified by MR and nMR at any timepoint and association with rwPFS (*n* = 31). **E,** Forest plot of the CPH model with RECIST included (CR/PR/SD vs. PD) as a covariate.

In the subset of patients with documented best overall RECIST outcome recorded at the 6-month appointment (*n* = 75), RECIST response correlated with rwPFS as expected (Fig. 4A). Stratification of these patients into MR and nMR groups showed similar rwPFS patterns as the RECIST categories of complete response (CR)/partial response (PR)/SD versus progressive disease (PD; Fig. 4B). Assessment of best percentage TF change (best MR) within the first 6 months showed trends consistent with RECIST responses (Fig. 4C). The median percentage change in TF was significantly different in between patients with CR/PR compared with those with PD (*T* test *P* = 0.002), and patients with MR were more likely to have improved RECIST best overall response, with MR highly enriched for CR/PR cases (*χ*^2^*P* = 0.0003). For patients classified as SD by RECIST, MR identified a subgroup of patients with significantly improved clinical outcomes [HR, 0.26 (95% CI, 0.09–0.72); *P* = 0.006; Fig. 4D]. Both ctDNA change and RECIST response were independently associated with time to rwPFS event after adjusting for both in a multivariate CPH model [HR, 0.11 (95% CI, 0.05–0.29), *P* < 0.005 and HR, 0.07 (95% CI, 0.03–0.19), *P* < 0.005, respectively], highlighting the additional value of ctDNA dynamics (Fig. 4E).

**Figure 5:**
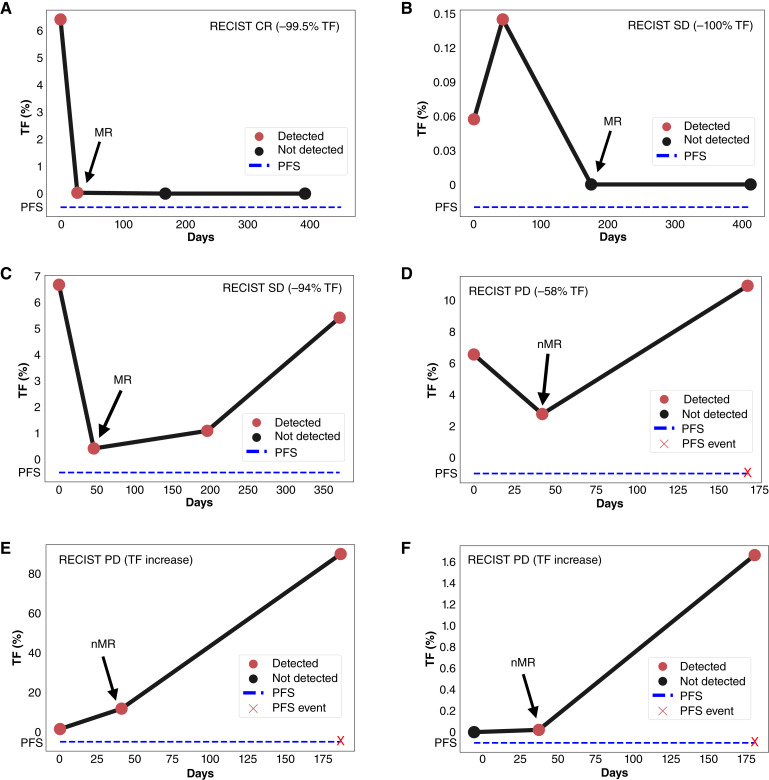
**A–F,** Representative patient examples demonstrating serial monitoring of TF for patients with (**A**) CR with head and neck squamous cell carcinoma; rwPFS = 15 months. **B,** SD with NSCLC; rwPFS = 13.77 months. **C,** SD with endometrial carcinoma; rwPFS = 12.37 months. **D,** PD with hepatocellular cancer; rwPFS = 5.6 months. **E,** PD with SCLC; rwPFS = 6.233 months. **F,** PD with NSCLC; rwPFS = 6.07 months; notably, TF was not detected at baseline.

Representative patient examples of longitudinal TF are shown in Fig. 5. A patient with a 6-month best RECIST response of CR had 99% decrease in TF at T1 and stayed below the LOQ through T3 (Fig. 5A). For patients with SD, one had an initial increase but subsequent 100% decrease at T2 which was sustained at T3 (delayed response) whereas the other had a large decrease at T1 (−94%) and then increase at T2 and further increase at T3 (Fig. 5B and C). Those with PD either had an initial decrease at T1 (−58%) and then increase at T2 or increase at both T1 and T2 (Fig. 5D–F). The final patient with PD was not detected at baseline but detectable at T1, which would be classified as nMR, and the patient then had a much larger increase by T2 (Fig. 5F). This final example demonstrates the validity of longitudinal monitoring in patients with no ctDNA detected at baseline.

## Discussion

This study demonstrated the clinical validity of longitudinal TF monitoring using a tissue-free, methylation-based assay in a real-world cohort of more than 500 patients with advanced cancers receiving ICI therapy. Patients with MR had significantly better outcomes than those without MR. Monitoring TF at multiple timepoints identified 21% more patients with MR compared with a single early assessment, and nMR status preceded documented progression events by a median of 3.03 months. Additionally, MR identified a subgroup of patients with RECIST SD who had significantly improved rwPFS, emphasizing the potential for TF to complement imaging-based assessments.

Previous studies using genomics-based assays have applied varying MR thresholds, including any decrease, 50% decrease, 90% decrease, and full clearance (12, 13, 19, 27, 35, 36). Studies by Bratman and colleagues (19), Zhang and colleagues (12), and Toledo and colleagues (37) evaluated MR in cohorts of 94, 171, and 90 patients, respectively, with solid tumors treated with ICI therapy. Bratman and colleagues (19) found associations between increases and decreases in ctDNA with outcomes, Zhang and colleagues (12) found that a 50% decrease in the mean variant allele fraction best associated with outcomes, and Toledo and colleagues (37) found that those with clearance had significant improvement in both PFS and OS. The present study is larger than these prior studies, and the real-world nature provided a robust setting to evaluate the impact of TF monitoring in SoC treatment. Consistent with prior research, our findings indicate that any TF reduction is significantly associated with improved outcomes and demonstrated a linear relationship between TF change and outcomes, with greater reductions linked to more favorable prognoses (12, 13, 19, 29, 38). The use of an ≥80% decrease threshold optimized the detection of responders while maintaining predictive strength.

An advantage of the tissue-free approach is that it requires only plasma samples, eliminating the need for tumor biopsies. This enables rapid, minimally invasive monitoring of tumor burden with a short turnaround time, facilitating real-time assessment of treatment response. Additionally, by leveraging thousands of methylation markers, this assay provides a highly sensitive and specific measure of tumor burden while minimizing confounding from clonal hematopoiesis, a limitation of genomic-based assays (39–41). This approach has a high success rate with minimal sample requirements and thus expands accessibility of ctDNA monitoring in diverse clinical settings.

For patients with advanced-stage cancer, early identification of treatment response or progression is critical for optimizing therapy. With a median lead time of 3.03 months before documented rwPFS events in this study, longitudinal TF monitoring provides a potential opportunity to adjust treatment strategies earlier. The data generated in this study have the potential to inform future studies to understand how changes in treatment strategies based on TF monitoring affect patient outcomes. Additionally, for patients experiencing side effects from treatment, early TF changes may help inform decisions about continuing or modifying treatment. Among those evaluated from longitudinal monitoring in this study, 27% (*n* = 143/521) experienced an irAE, including 26% (*n* = 37/143) of those experiencing a severe irAE. Thus, the lead time of 3 months is clinically meaningful as 12 weeks could involve four or more infusions of ineffective ICI therapy risking toxicity, increasing costs, and potentially reducing the effectiveness of subsequent rounds of treatment because of the delay in alternative treatment initiation.

TF monitoring has potential applications in drug development (39, 42–45) and clinical trial design, particularly in identifying patients who may benefit from therapy escalation. ctDNA has also been proposed as an intermediate endpoint (46–48), though broader implementation has been limited by a lack of randomized trials incorporating ctDNA alongside established endpoints. This study demonstrated that baseline TF and on-treatment TF changes are independent factors, supporting their use in stratifying patients in clinical trials and assessing drug efficacy.

This real-world study has some limitations. Ideally, as an important predictor of survival outcomes related to ICI therapy, Eastern Cooperative Oncology Group status would have been included in demographics and CPH models. However, that information was not collected as part of the RADIOHEAD study and therefore could not be incorporated. rwPFS and rwOS events were physician-reported and may be underreported or approximated. Patients who progressed before cycle 3, day 1 visit were not evaluated for longitudinal monitoring because of the absence of follow-up samples. Additionally, study follow-up was limited to 15 months, restricting long-term OS assessment. Nonetheless, MR provided insight into treatment response in cases in which imaging data were unavailable. Given the timing and frequency of blood collection in this study, the lead time of nMR to clinical progression may be underestimated. Future studies with more frequent blood collections are needed to clarify this. Whereas samples were to be collected at baseline, cycle 3 day 1, 6 months, and 12 months, additional samples were drawn at the time of irAE incidence. As a result, and due to other variation due to the real-world setting, samples were collected at varying timepoints. For the purposes of the longitudinal monitoring analyses, all samples collected ≥15 days after treatment initiation were grouped into three ranges of time using the first available sample within that range for analysis. This approach retains the real-world nature of the study and maximizes generalizability to real-world clinical practice.

Whereas we demonstrated that patients with increasing ctDNA had shorter median rwPFS, not all patients with rwPFS events had increasing ctDNA. This may reflect the overall low ctDNA levels and favorable prognosis of the cohort, as well as some degree of incomplete real-world data. Similarly, a subset of patients with RECIST-defined PD as their best response at 6 months showed ≥80% reductions in ctDNA (MR) within that timeframe. This discordance could reflect pseudoprogression or delayed response, though confirmation was not possible given the limitations of this dataset. As such, future studies with more frequent blood collections, along with temporally matched imaging and detailed clinical follow-up, will be critical to further clarify molecular progression. Despite these limitations, a strong and statistically significant association was observed between TF percentage change and real-world outcomes.

A strength of this study is its diverse patient population receiving various ICI regimens closely reflecting real-world clinical practice. Future research will include assessment of TF in other therapeutic settings and earlier-stage cancers, as well as the development of methylation-based predictors of immunotherapy efficacy and toxicity. Additionally, future work will also explore how TF integrates with complementary molecular datasets from the RADIOHEAD study, such as high-dimensional flow cytometry, transcriptomics, and serum proteomics.

In summary, serial TF monitoring using a methylation-based assay demonstrated clinically meaningful and statistically significant associations with rwPFS and rwOS in this large cohort of more than 500 patients receiving SoC ICI therapy. Monitoring TF across multiple timepoints improved predictive value for rwPFS over single early on-treatment timepoint assessments, and nMR preceded clinical progression with a median lead time of 3.03 months. Prospective trials are needed to validate the clinical utility of MR using methylation-based TF in diverse treatment settings.

## Supplementary Material

Supplementary Table S1 ICI Therapy Type per Cancer Type

Supplementary Table S2 Methylation TF Results by Timepoint

Supplementary Figure S1 Timing of sample collection

Supplementary Table S3 Successful samples by cancer type and timepoint

Supplementary Figure S2 Swimmers Plots by cancer type

Supplementary Figure S3 Baseline TF detection is prognostic but TF change is predictive of outcome

Supplementary Table S4 Quartiles of baseline TF across cancer type

Supplementary Figure S4 Forest Plot analysis for percentage TF change threshold determination

Supplementary Table S5 Longitudinal assessment of TF predicts ICI benefit

Supplementary Figure S5 Real-world outcomes of patients with TF below LOQ at both timepoints

Supplementary Table S6 Sensitivity analysis used to demonstrate impact of time ranges on longitudinal monitoring analysis

Supplementary Table S7 Longitudinal assessment of TF predicts ICI benefit in individual cancers
