# Supplementary material for: Methylation-Based ctDNA Tumor Fraction Changes Predict Long-Term Clinical Benefit From Immune Checkpoint Inhibitors in RADIOHEAD, a Real-World Pan-Cancer Study
Source: Cancer Res Commun. 2025 Aug 20;5(8):1384–95. doi: 10.1158/2767-9764.CRC-25-0151 (PMC12365632; doi:10.1158/2767-9764.CRC-25-0151)
Supplement: Supplementary Table S1 — ICI Therapy Type per Cancer Type [file crc-25-0151_supplementary_table_s1_suppst1.pptx]

## Slide 1
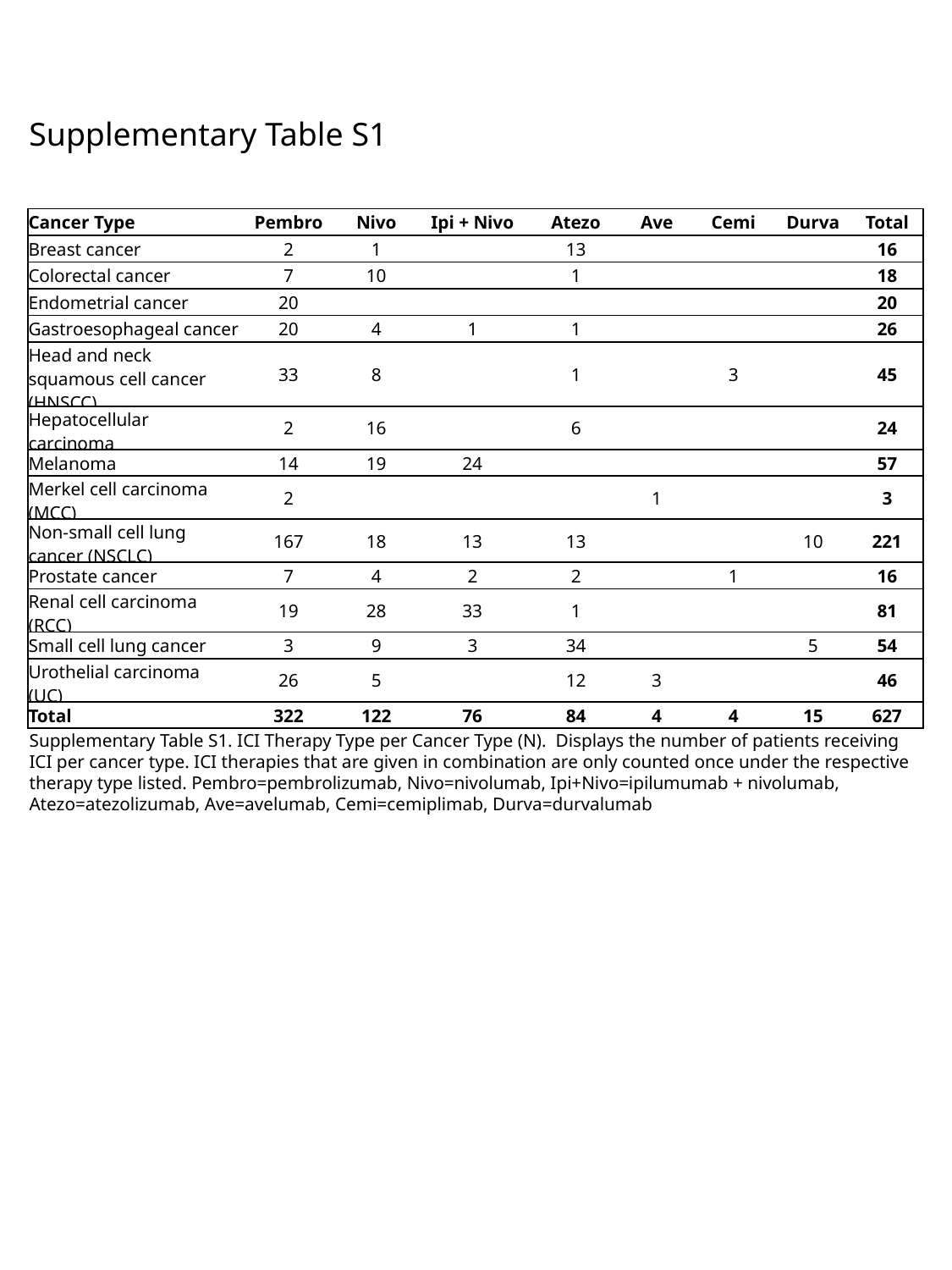

Supplementary Table S1
| Cancer Type | Pembro | Nivo | Ipi + Nivo | Atezo | Ave | Cemi | Durva | Total |
| --- | --- | --- | --- | --- | --- | --- | --- | --- |
| Breast cancer | 2 | 1 | | 13 | | | | 16 |
| Colorectal cancer | 7 | 10 | | 1 | | | | 18 |
| Endometrial cancer | 20 | | | | | | | 20 |
| Gastroesophageal cancer | 20 | 4 | 1 | 1 | | | | 26 |
| Head and neck squamous cell cancer (HNSCC) | 33 | 8 | | 1 | | 3 | | 45 |
| Hepatocellular carcinoma | 2 | 16 | | 6 | | | | 24 |
| Melanoma | 14 | 19 | 24 | | | | | 57 |
| Merkel cell carcinoma (MCC) | 2 | | | | 1 | | | 3 |
| Non-small cell lung cancer (NSCLC) | 167 | 18 | 13 | 13 | | | 10 | 221 |
| Prostate cancer | 7 | 4 | 2 | 2 | | 1 | | 16 |
| Renal cell carcinoma (RCC) | 19 | 28 | 33 | 1 | | | | 81 |
| Small cell lung cancer | 3 | 9 | 3 | 34 | | | 5 | 54 |
| Urothelial carcinoma (UC) | 26 | 5 | | 12 | 3 | | | 46 |
| Total | 322 | 122 | 76 | 84 | 4 | 4 | 15 | 627 |
Supplementary Table S1. ICI Therapy Type per Cancer Type (N). Displays the number of patients receiving ICI per cancer type. ICI therapies that are given in combination are only counted once under the respective therapy type listed. Pembro=pembrolizumab, Nivo=nivolumab, Ipi+Nivo=ipilumumab + nivolumab, Atezo=atezolizumab, Ave=avelumab, Cemi=cemiplimab, Durva=durvalumab
