# Supplementary material for: Methylation-Based ctDNA Tumor Fraction Changes Predict Long-Term Clinical Benefit From Immune Checkpoint Inhibitors in RADIOHEAD, a Real-World Pan-Cancer Study
Source: Cancer Res Commun. 2025 Aug 20;5(8):1384–95. doi: 10.1158/2767-9764.CRC-25-0151 (PMC12365632; doi:10.1158/2767-9764.CRC-25-0151)
Supplement: Supplementary Figure S1 — Timing of sample collection [file crc-25-0151_supplementary_figure_s1_suppsf1.pptx]

## Slide 1
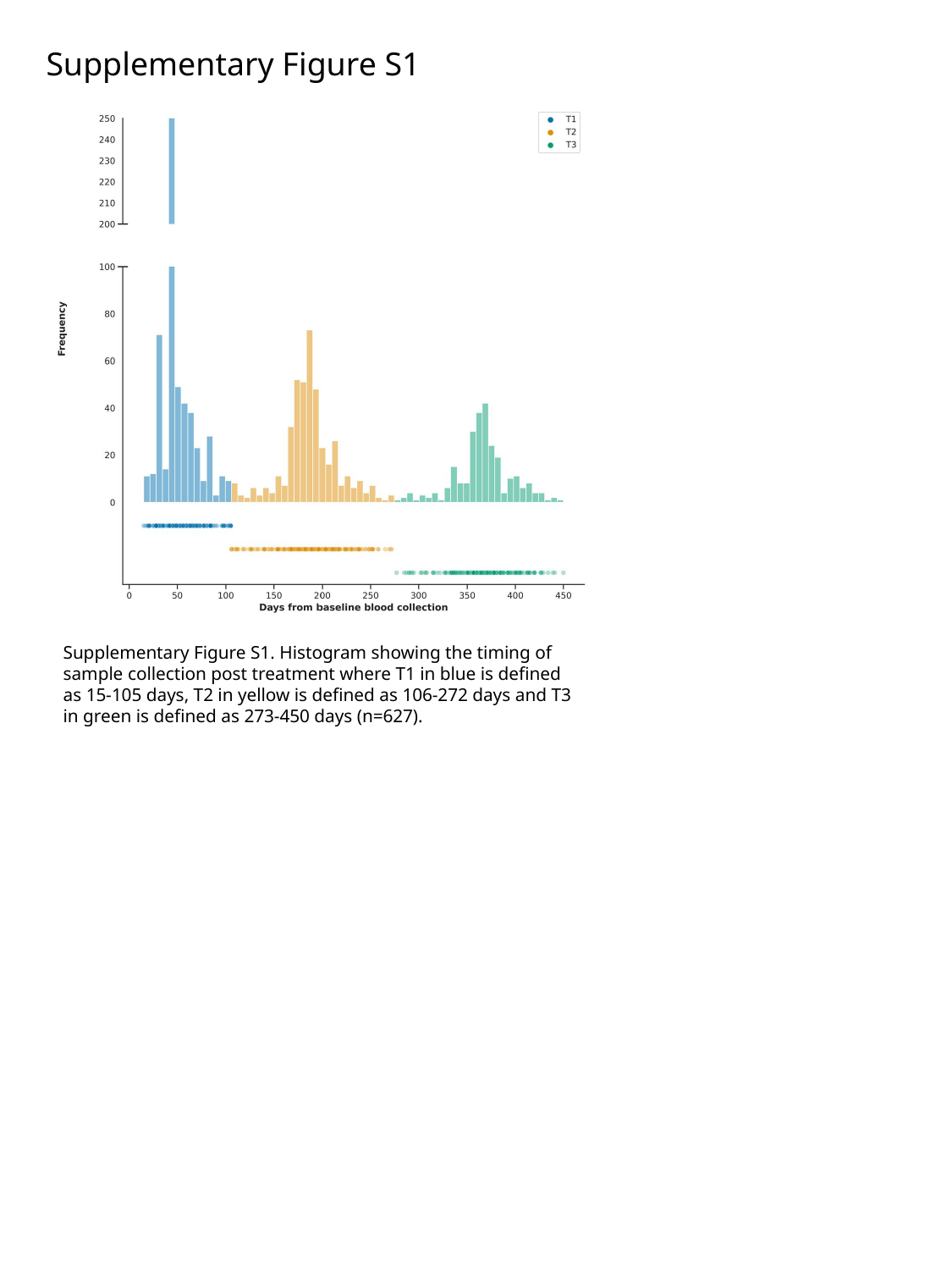

Supplementary Figure S1
Supplementary Figure S1. Histogram showing the timing of sample collection post treatment where T1 in blue is defined as 15-105 days, T2 in yellow is defined as 106-272 days and T3 in green is defined as 273-450 days (n=627).
