# Supplementary material for: Methylation-Based ctDNA Tumor Fraction Changes Predict Long-Term Clinical Benefit From Immune Checkpoint Inhibitors in RADIOHEAD, a Real-World Pan-Cancer Study
Source: Cancer Res Commun. 2025 Aug 20;5(8):1384–95. doi: 10.1158/2767-9764.CRC-25-0151 (PMC12365632; doi:10.1158/2767-9764.CRC-25-0151)
Supplement: Supplementary Table S3 — Successful samples by cancer type and timepoint [file crc-25-0151_supplementary_table_s3_suppst3.pptx]

## Slide 1
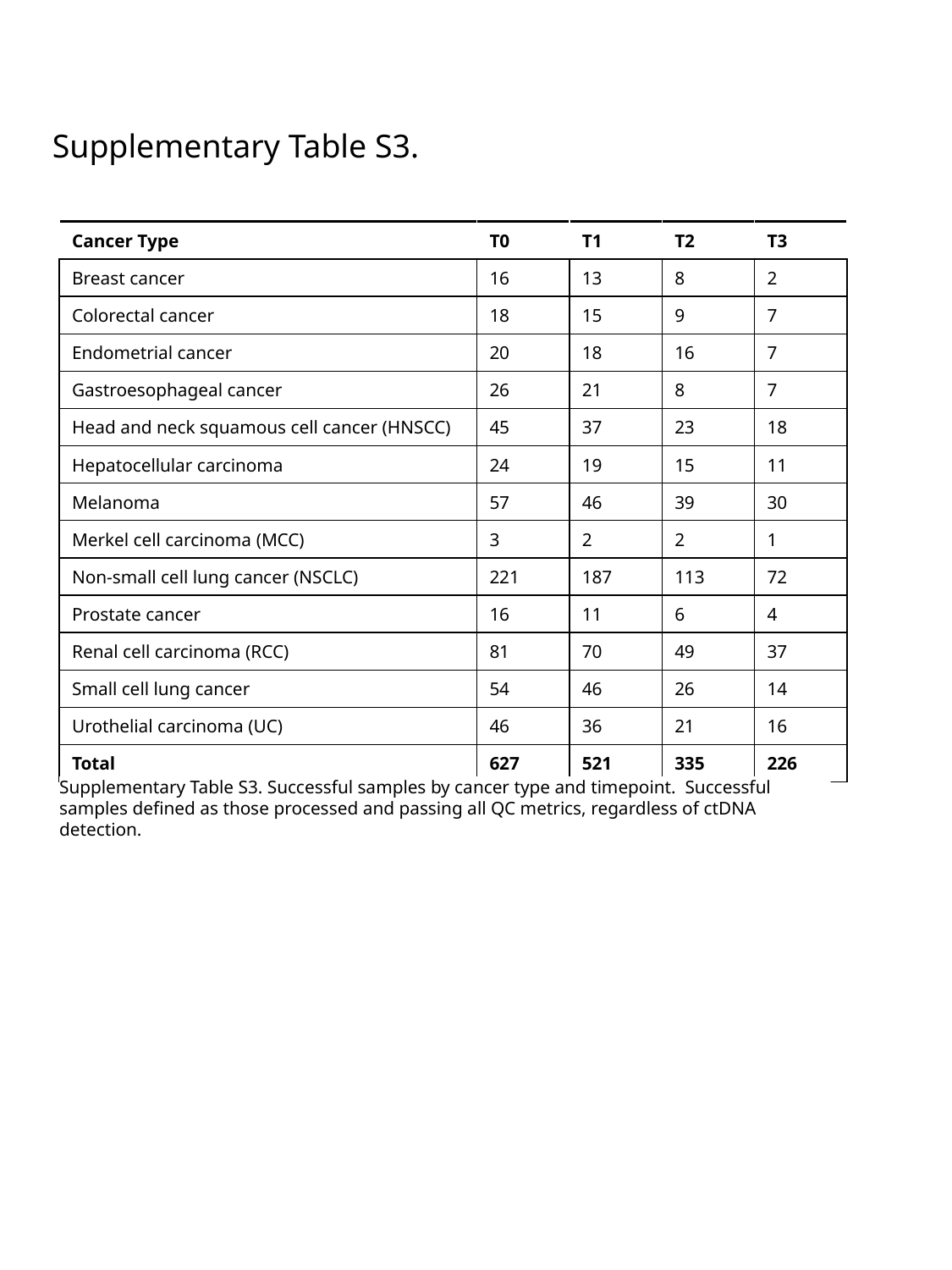

Supplementary Table S3.
| Cancer Type | T0 | T1 | T2 | T3 |
| --- | --- | --- | --- | --- |
| Breast cancer | 16 | 13 | 8 | 2 |
| Colorectal cancer | 18 | 15 | 9 | 7 |
| Endometrial cancer | 20 | 18 | 16 | 7 |
| Gastroesophageal cancer | 26 | 21 | 8 | 7 |
| Head and neck squamous cell cancer (HNSCC) | 45 | 37 | 23 | 18 |
| Hepatocellular carcinoma | 24 | 19 | 15 | 11 |
| Melanoma | 57 | 46 | 39 | 30 |
| Merkel cell carcinoma (MCC) | 3 | 2 | 2 | 1 |
| Non-small cell lung cancer (NSCLC) | 221 | 187 | 113 | 72 |
| Prostate cancer | 16 | 11 | 6 | 4 |
| Renal cell carcinoma (RCC) | 81 | 70 | 49 | 37 |
| Small cell lung cancer | 54 | 46 | 26 | 14 |
| Urothelial carcinoma (UC) | 46 | 36 | 21 | 16 |
| Total | 627 | 521 | 335 | 226 |
Supplementary Table S3. Successful samples by cancer type and timepoint.  Successful samples defined as those processed and passing all QC metrics, regardless of ctDNA detection.
