# Supplementary material for: Methylation-Based ctDNA Tumor Fraction Changes Predict Long-Term Clinical Benefit From Immune Checkpoint Inhibitors in RADIOHEAD, a Real-World Pan-Cancer Study
Source: Cancer Res Commun. 2025 Aug 20;5(8):1384–95. doi: 10.1158/2767-9764.CRC-25-0151 (PMC12365632; doi:10.1158/2767-9764.CRC-25-0151)
Supplement: Supplementary Figure S2 — Swimmers Plots by cancer type [file crc-25-0151_supplementary_figure_s2_suppsf2.pptx]

## Slide 1
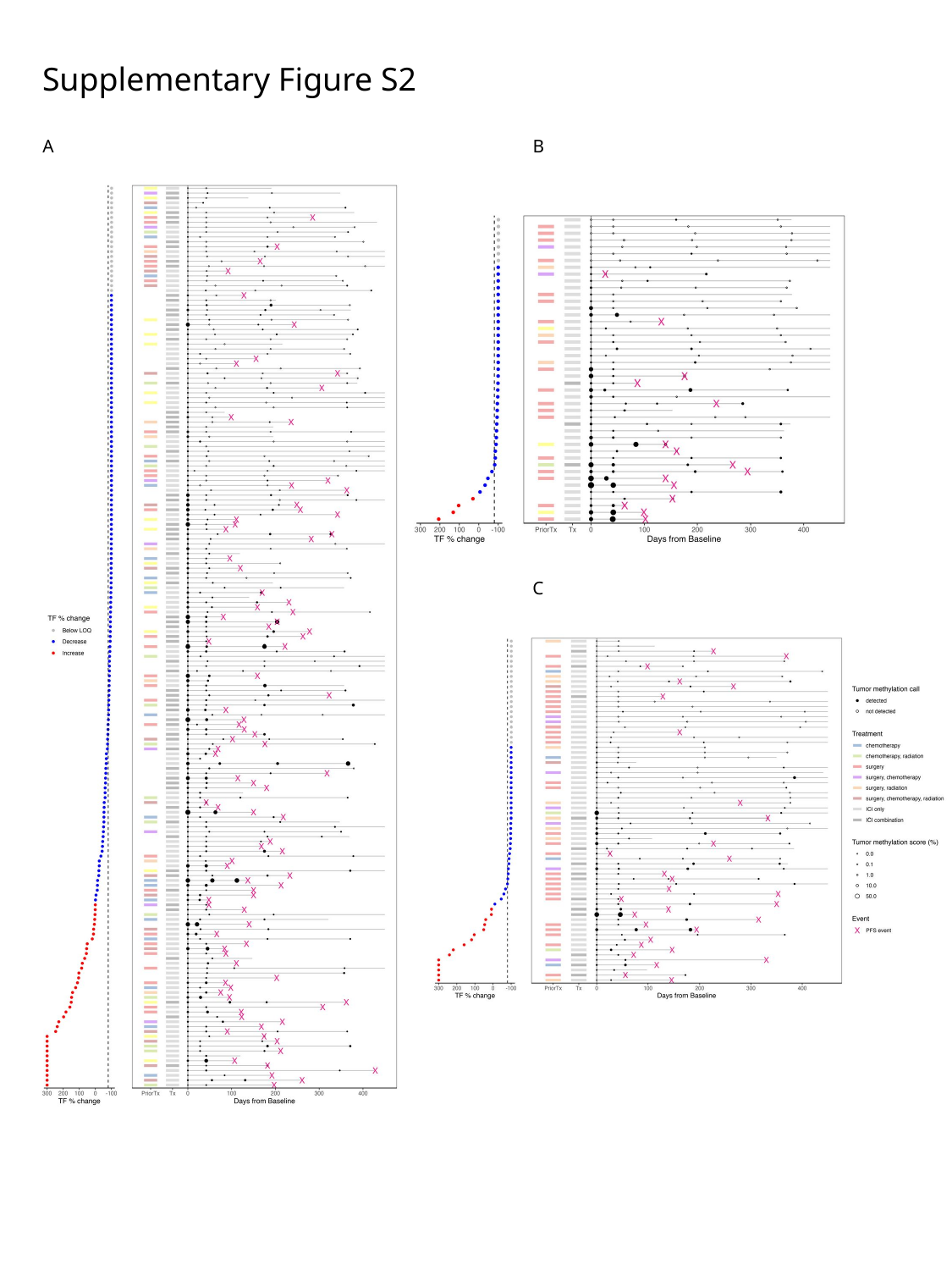

Supplementary Figure S2
A
B
C

## Slide 2
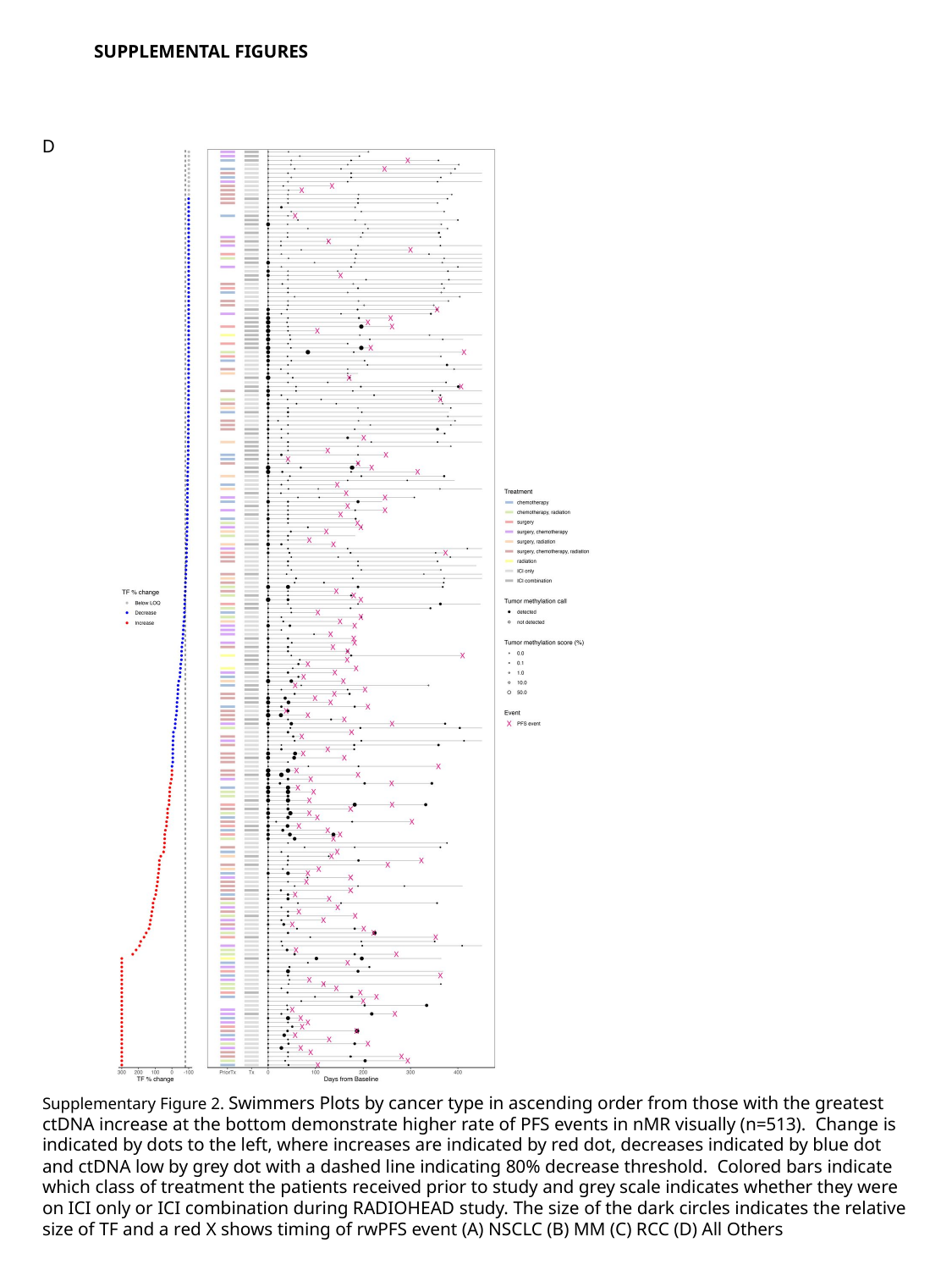

SUPPLEMENTAL FIGURES
D
Supplementary Figure 2. Swimmers Plots by cancer type in ascending order from those with the greatest ctDNA increase at the bottom demonstrate higher rate of PFS events in nMR visually (n=513).  Change is indicated by dots to the left, where increases are indicated by red dot, decreases indicated by blue dot and ctDNA low by grey dot with a dashed line indicating 80% decrease threshold.  Colored bars indicate which class of treatment the patients received prior to study and grey scale indicates whether they were on ICI only or ICI combination during RADIOHEAD study. The size of the dark circles indicates the relative size of TF and a red X shows timing of rwPFS event (A) NSCLC (B) MM (C) RCC (D) All Others
