# Supplementary material for: Methylation-Based ctDNA Tumor Fraction Changes Predict Long-Term Clinical Benefit From Immune Checkpoint Inhibitors in RADIOHEAD, a Real-World Pan-Cancer Study
Source: Cancer Res Commun. 2025 Aug 20;5(8):1384–95. doi: 10.1158/2767-9764.CRC-25-0151 (PMC12365632; doi:10.1158/2767-9764.CRC-25-0151)
Supplement: Supplementary Figure S3 — Baseline TF detection is prognostic but TF change is predictive of outcome [file crc-25-0151_supplementary_figure_s3_suppsf3.pptx]

## Slide 1
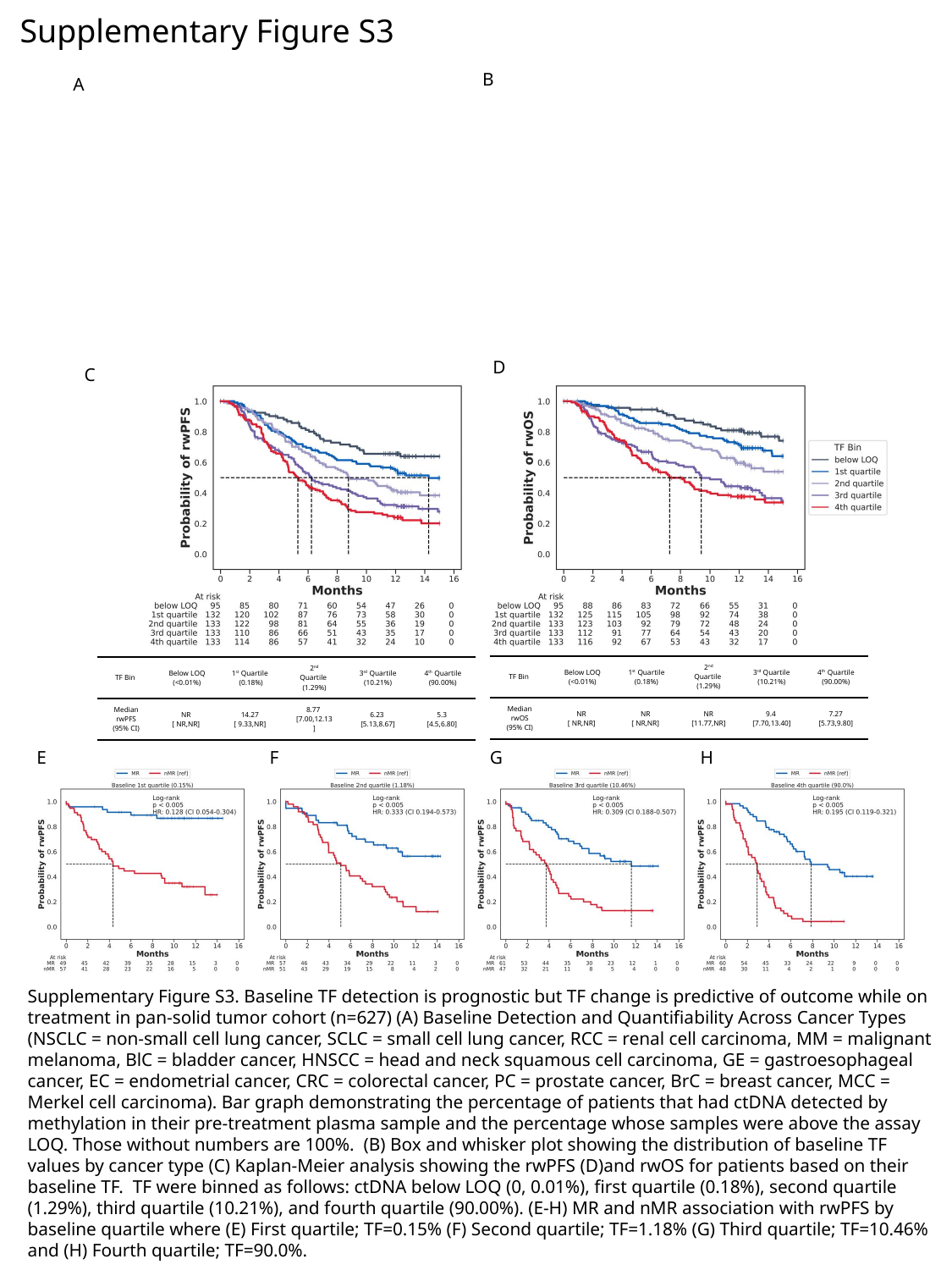

Supplementary Figure S3
B
A
D
C
| TF Bin | Below LOQ (<0.01%) | 1st Quartile (0.18%) | 2nd Quartile (1.29%) | 3rd Quartile (10.21%) | 4th Quartile (90.00%) |
| --- | --- | --- | --- | --- | --- |
| Median rwOS (95% CI) | NR [ NR,NR] | NR [ NR,NR] | NR [11.77,NR] | 9.4 [7.70,13.40] | 7.27 [5.73,9.80] |
| TF Bin | Below LOQ (<0.01%) | 1st Quartile (0.18%) | 2nd Quartile (1.29%) | 3rd Quartile (10.21%) | 4th Quartile (90.00%) |
| --- | --- | --- | --- | --- | --- |
| Median rwPFS (95% CI) | NR [ NR,NR] | 14.27 [ 9.33,NR] | 8.77 [7.00,12.13] | 6.23 [5.13,8.67] | 5.3 [4.5,6.80] |
F
H
E
G
Supplementary Figure S3. Baseline TF detection is prognostic but TF change is predictive of outcome while on treatment in pan-solid tumor cohort (n=627) (A) Baseline Detection and Quantifiability Across Cancer Types (NSCLC = non-small cell lung cancer, SCLC = small cell lung cancer, RCC = renal cell carcinoma, MM = malignant melanoma, BlC = bladder cancer, HNSCC = head and neck squamous cell carcinoma, GE = gastroesophageal cancer, EC = endometrial cancer, CRC = colorectal cancer, PC = prostate cancer, BrC = breast cancer, MCC = Merkel cell carcinoma). Bar graph demonstrating the percentage of patients that had ctDNA detected by methylation in their pre-treatment plasma sample and the percentage whose samples were above the assay LOQ. Those without numbers are 100%.  (B) Box and whisker plot showing the distribution of baseline TF values by cancer type (C) Kaplan-Meier analysis showing the rwPFS (D)and rwOS for patients based on their baseline TF.  TF were binned as follows: ctDNA below LOQ (0, 0.01%), first quartile (0.18%), second quartile (1.29%), third quartile (10.21%), and fourth quartile (90.00%). (E-H) MR and nMR association with rwPFS by baseline quartile where (E) First quartile; TF=0.15% (F) Second quartile; TF=1.18% (G) Third quartile; TF=10.46% and (H) Fourth quartile; TF=90.0%.
