# Supplementary material for: Methylation-Based ctDNA Tumor Fraction Changes Predict Long-Term Clinical Benefit From Immune Checkpoint Inhibitors in RADIOHEAD, a Real-World Pan-Cancer Study
Source: Cancer Res Commun. 2025 Aug 20;5(8):1384–95. doi: 10.1158/2767-9764.CRC-25-0151 (PMC12365632; doi:10.1158/2767-9764.CRC-25-0151)
Supplement: Supplementary Table S4 — Quartiles of baseline TF across cancer type [file crc-25-0151_supplementary_table_s4_suppst4.pptx]

## Slide 1
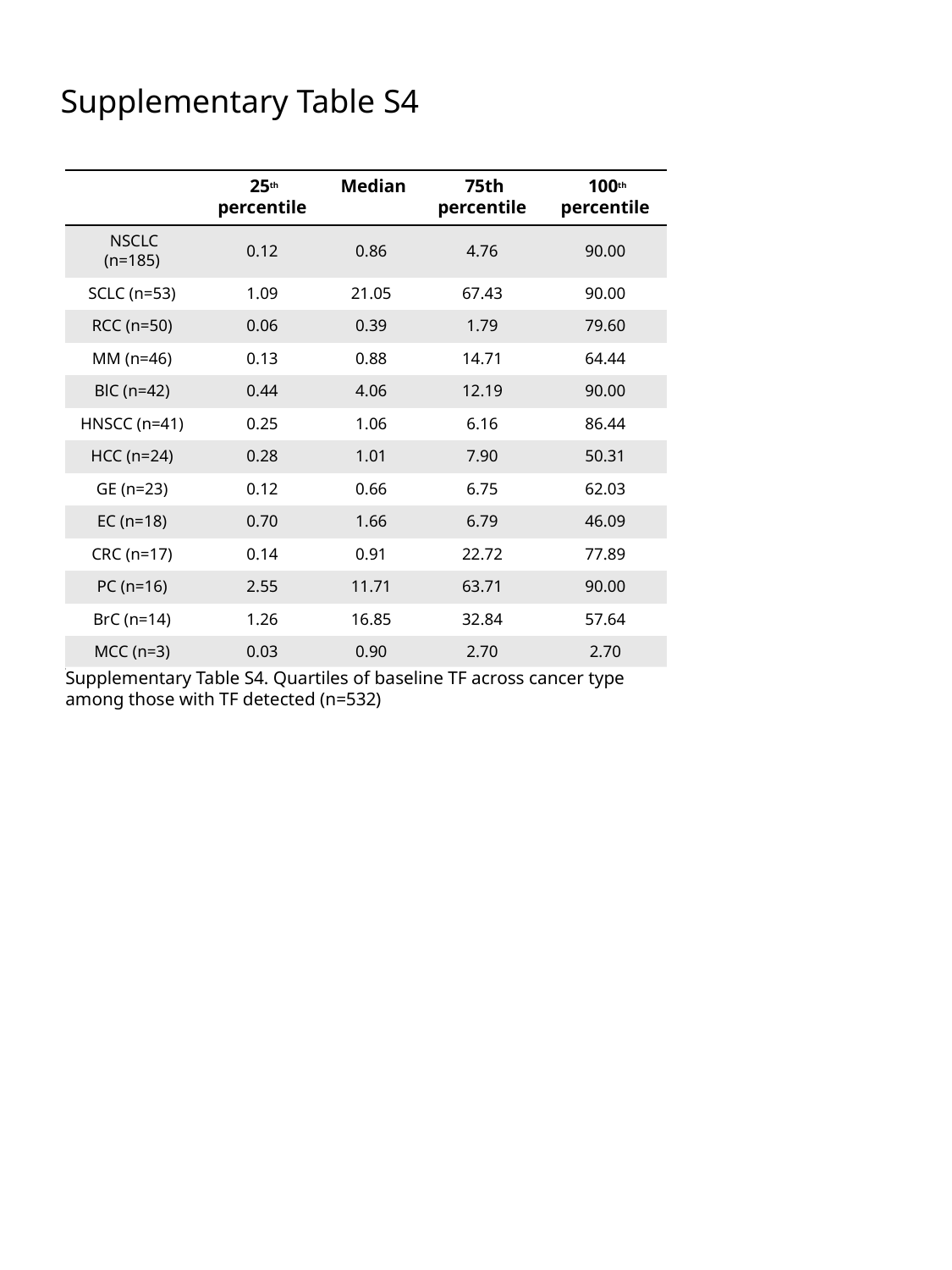

Supplementary Table S4
| | 25th percentile | Median | 75th percentile | 100th percentile |
| --- | --- | --- | --- | --- |
| NSCLC (n=185) | 0.12 | 0.86 | 4.76 | 90.00 |
| SCLC (n=53) | 1.09 | 21.05 | 67.43 | 90.00 |
| RCC (n=50) | 0.06 | 0.39 | 1.79 | 79.60 |
| MM (n=46) | 0.13 | 0.88 | 14.71 | 64.44 |
| BlC (n=42) | 0.44 | 4.06 | 12.19 | 90.00 |
| HNSCC (n=41) | 0.25 | 1.06 | 6.16 | 86.44 |
| HCC (n=24) | 0.28 | 1.01 | 7.90 | 50.31 |
| GE (n=23) | 0.12 | 0.66 | 6.75 | 62.03 |
| EC (n=18) | 0.70 | 1.66 | 6.79 | 46.09 |
| CRC (n=17) | 0.14 | 0.91 | 22.72 | 77.89 |
| PC (n=16) | 2.55 | 11.71 | 63.71 | 90.00 |
| BrC (n=14) | 1.26 | 16.85 | 32.84 | 57.64 |
| MCC (n=3) | 0.03 | 0.90 | 2.70 | 2.70 |
Supplementary Table S4. Quartiles of baseline TF across cancer type among those with TF detected (n=532)
