# Supplementary material for: Methylation-Based ctDNA Tumor Fraction Changes Predict Long-Term Clinical Benefit From Immune Checkpoint Inhibitors in RADIOHEAD, a Real-World Pan-Cancer Study
Source: Cancer Res Commun. 2025 Aug 20;5(8):1384–95. doi: 10.1158/2767-9764.CRC-25-0151 (PMC12365632; doi:10.1158/2767-9764.CRC-25-0151)
Supplement: Supplementary Figure S4 — Forest Plot analysis for percentage TF change threshold determination [file crc-25-0151_supplementary_figure_s4_suppsf4.pptx]

## Slide 1
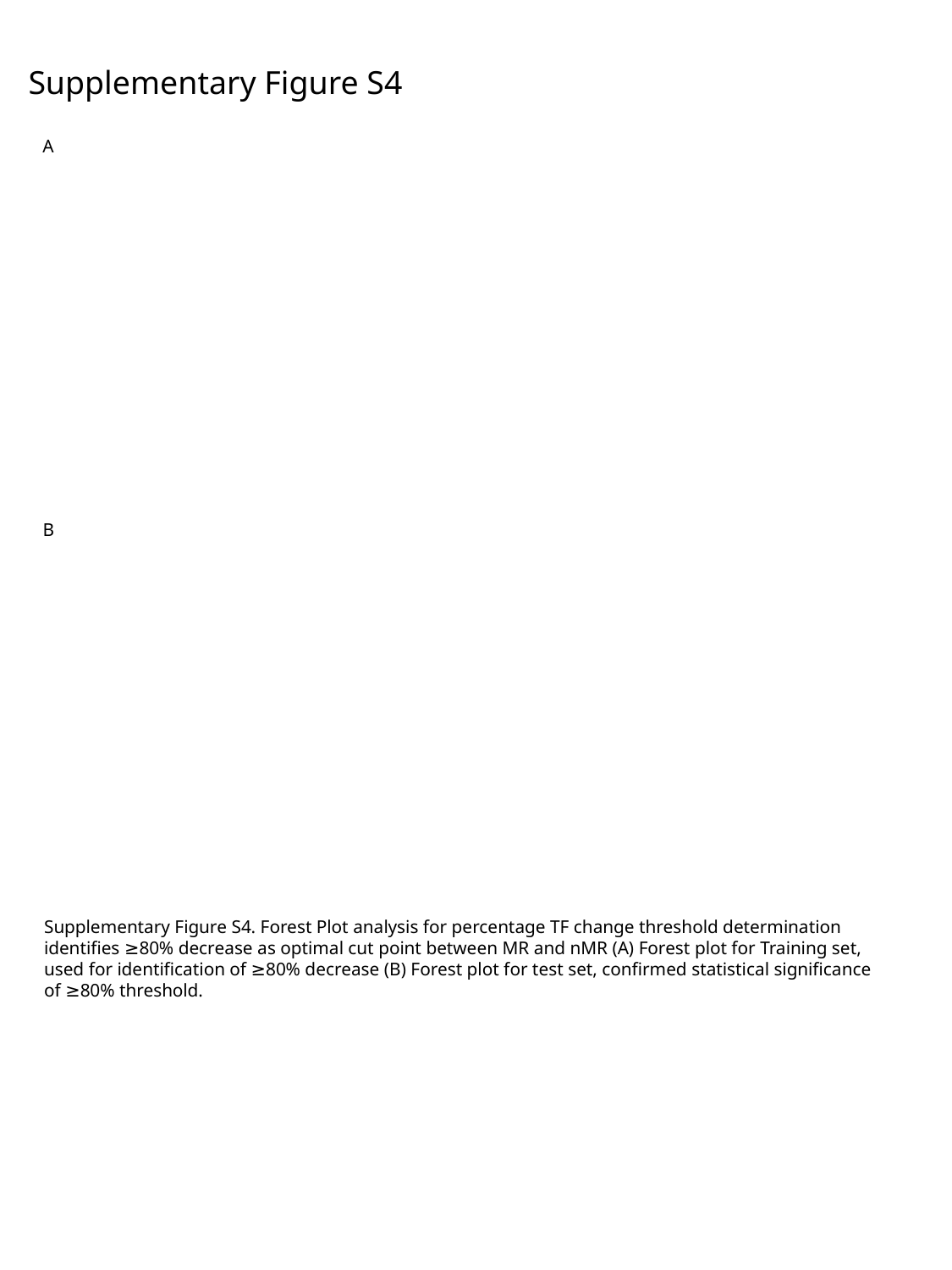

Supplementary Figure S4
A
B
Supplementary Figure S4. Forest Plot analysis for percentage TF change threshold determination identifies ≥80% decrease as optimal cut point between MR and nMR (A) Forest plot for Training set, used for identification of ≥80% decrease (B) Forest plot for test set, confirmed statistical significance of ≥80% threshold.
