# Supplementary material for: Methylation-Based ctDNA Tumor Fraction Changes Predict Long-Term Clinical Benefit From Immune Checkpoint Inhibitors in RADIOHEAD, a Real-World Pan-Cancer Study
Source: Cancer Res Commun. 2025 Aug 20;5(8):1384–95. doi: 10.1158/2767-9764.CRC-25-0151 (PMC12365632; doi:10.1158/2767-9764.CRC-25-0151)
Supplement: Supplementary Table S5 — Longitudinal assessment of TF predicts ICI benefit [file crc-25-0151_supplementary_table_s5_suppst5.pptx]

## Slide 1
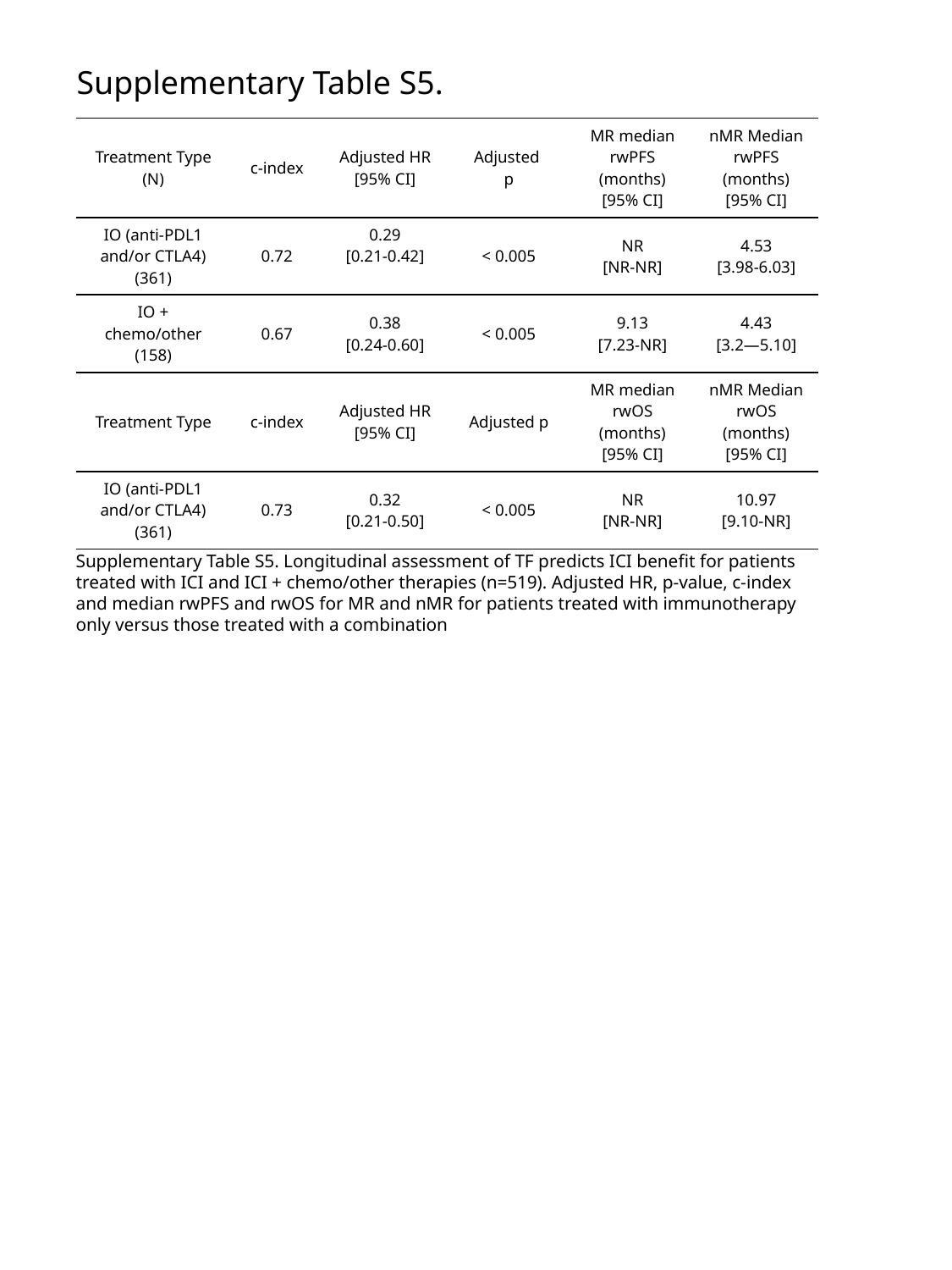

Supplementary Table S5.
| Treatment Type (N) | c-index | Adjusted HR [95% CI] | Adjusted p | MR median rwPFS (months) [95% CI] | nMR Median rwPFS (months) [95% CI] |
| --- | --- | --- | --- | --- | --- |
| IO (anti-PDL1 and/or CTLA4) (361) | 0.72 | 0.29 [0.21-0.42] | < 0.005 | NR [NR-NR] | 4.53 [3.98-6.03] |
| IO + chemo/other (158) | 0.67 | 0.38 [0.24-0.60] | < 0.005 | 9.13 [7.23-NR] | 4.43 [3.2—5.10] |
| Treatment Type | c-index | Adjusted HR [95% CI] | Adjusted p | MR median rwOS (months) [95% CI] | nMR Median rwOS (months) [95% CI] |
| IO (anti-PDL1 and/or CTLA4) (361) | 0.73 | 0.32 [0.21-0.50] | < 0.005 | NR [NR-NR] | 10.97 [9.10-NR] |
| IO + chemo/other (158) | 0.69 | 0.40 [0.24-0.68] | < 0.005 | NR [9.53-NR] | 7.6 [5.70-11.27] |
Supplementary Table S5. Longitudinal assessment of TF predicts ICI benefit for patients treated with ICI and ICI + chemo/other therapies (n=519). Adjusted HR, p-value, c-index and median rwPFS and rwOS for MR and nMR for patients treated with immunotherapy only versus those treated with a combination
