# Supplementary material for: Methylation-Based ctDNA Tumor Fraction Changes Predict Long-Term Clinical Benefit From Immune Checkpoint Inhibitors in RADIOHEAD, a Real-World Pan-Cancer Study
Source: Cancer Res Commun. 2025 Aug 20;5(8):1384–95. doi: 10.1158/2767-9764.CRC-25-0151 (PMC12365632; doi:10.1158/2767-9764.CRC-25-0151)
Supplement: Supplementary Figure S5 — Real-world outcomes of patients with TF below LOQ at both timepoints [file crc-25-0151_supplementary_figure_s5_suppsf5.pptx]

## Slide 1
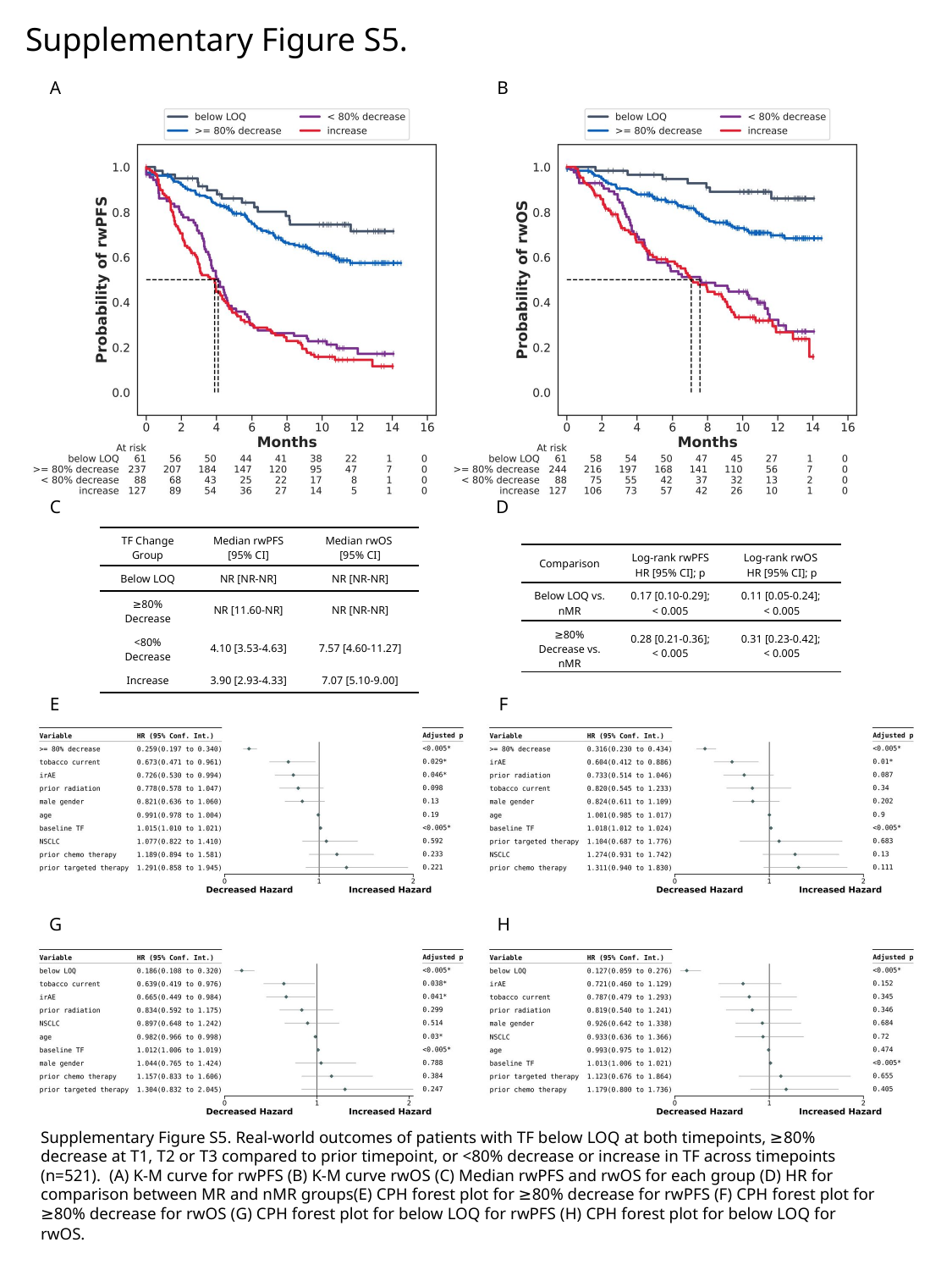

Supplementary Figure S5.
B
A
D
C
| TF Change Group | Median rwPFS [95% CI] | Median rwOS [95% CI] |
| --- | --- | --- |
| Below LOQ | NR [NR-NR] | NR [NR-NR] |
| ≥80% Decrease | NR [11.60-NR] | NR [NR-NR] |
| <80% Decrease | 4.10 [3.53-4.63] | 7.57 [4.60-11.27] |
| Increase | 3.90 [2.93-4.33] | 7.07 [5.10-9.00] |
| Comparison | Log-rank rwPFS HR [95% CI]; p | Log-rank rwOS HR [95% CI]; p |
| --- | --- | --- |
| Below LOQ vs. nMR | 0.17 [0.10-0.29]; < 0.005 | 0.11 [0.05-0.24]; < 0.005 |
| ≥80% Decrease vs. nMR | 0.28 [0.21-0.36]; < 0.005 | 0.31 [0.23-0.42]; < 0.005 |
F
E
H
G
Supplementary Figure S5. Real-world outcomes of patients with TF below LOQ at both timepoints, ≥80% decrease at T1, T2 or T3 compared to prior timepoint, or <80% decrease or increase in TF across timepoints (n=521).  (A) K-M curve for rwPFS (B) K-M curve rwOS (C) Median rwPFS and rwOS for each group (D) HR for comparison between MR and nMR groups(E) CPH forest plot for ≥80% decrease for rwPFS (F) CPH forest plot for ≥80% decrease for rwOS (G) CPH forest plot for below LOQ for rwPFS (H) CPH forest plot for below LOQ for rwOS.
