# Supplementary material for: Methylation-Based ctDNA Tumor Fraction Changes Predict Long-Term Clinical Benefit From Immune Checkpoint Inhibitors in RADIOHEAD, a Real-World Pan-Cancer Study
Source: Cancer Res Commun. 2025 Aug 20;5(8):1384–95. doi: 10.1158/2767-9764.CRC-25-0151 (PMC12365632; doi:10.1158/2767-9764.CRC-25-0151)
Supplement: Supplementary Table S6 — Sensitivity analysis used to demonstrate impact of time ranges on longitudinal monitoring analysis [file crc-25-0151_supplementary_table_s6_suppst6.pptx]

## Slide 1
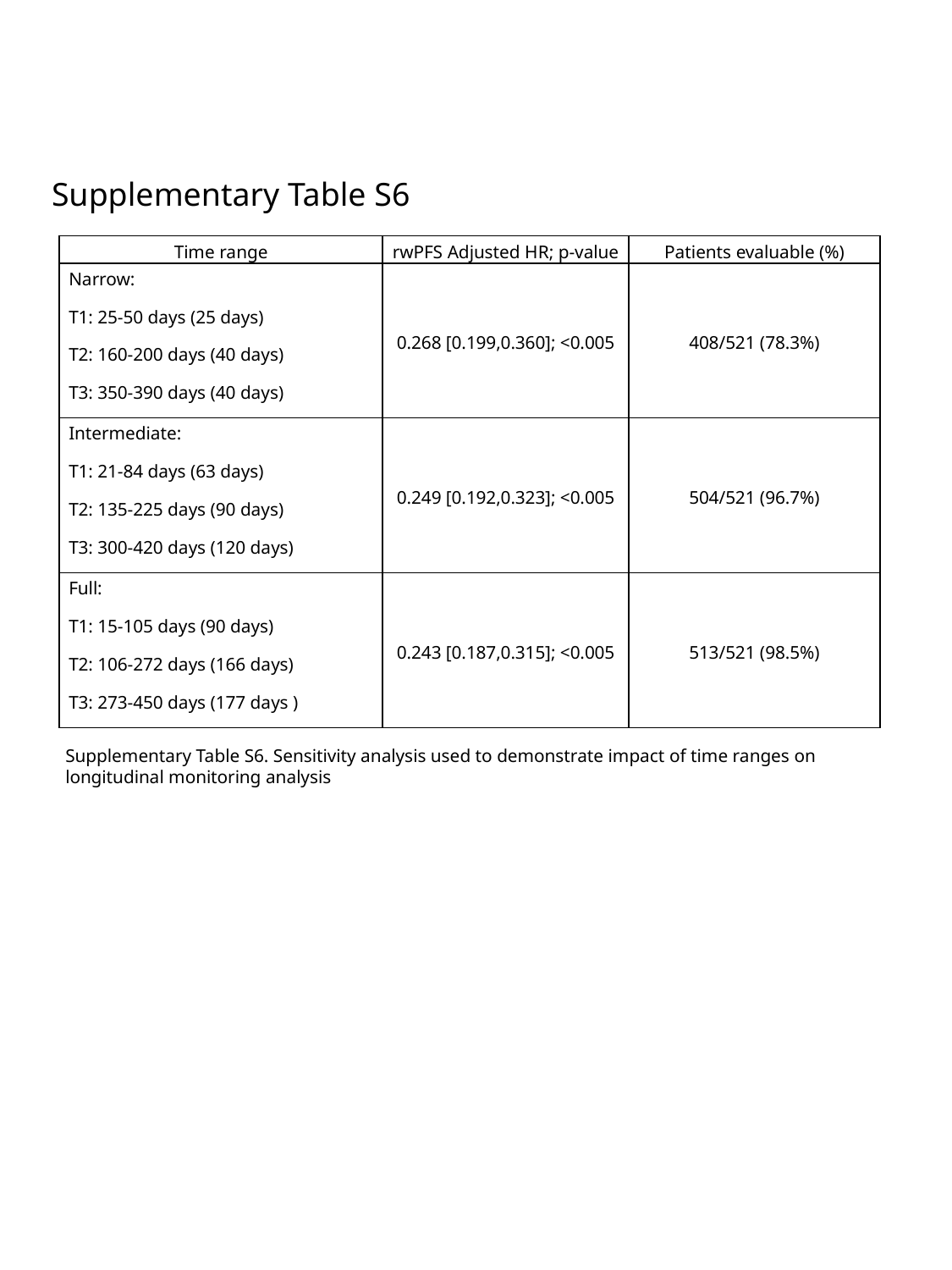

Supplementary Table S6
| Time range | rwPFS Adjusted HR; p-value | Patients evaluable (%) |
| --- | --- | --- |
| Narrow: T1: 25-50 days (25 days) T2: 160-200 days (40 days) T3: 350-390 days (40 days) | 0.268 [0.199,0.360]; <0.005 | 408/521 (78.3%) |
| Intermediate: T1: 21-84 days (63 days) T2: 135-225 days (90 days) T3: 300-420 days (120 days) | 0.249 [0.192,0.323]; <0.005 | 504/521 (96.7%) |
| Full: T1: 15-105 days (90 days) T2: 106-272 days (166 days) T3: 273-450 days (177 days ) | 0.243 [0.187,0.315]; <0.005 | 513/521 (98.5%) |
Supplementary Table S6. Sensitivity analysis used to demonstrate impact of time ranges on longitudinal monitoring analysis
