# Supplementary material for: Methylation-Based ctDNA Tumor Fraction Changes Predict Long-Term Clinical Benefit From Immune Checkpoint Inhibitors in RADIOHEAD, a Real-World Pan-Cancer Study
Source: Cancer Res Commun. 2025 Aug 20;5(8):1384–95. doi: 10.1158/2767-9764.CRC-25-0151 (PMC12365632; doi:10.1158/2767-9764.CRC-25-0151)
Supplement: Supplementary Table S7 — Longitudinal assessment of TF predicts ICI benefit in individual cancers [file crc-25-0151_supplementary_table_s7_suppst7.pptx]

## Slide 1
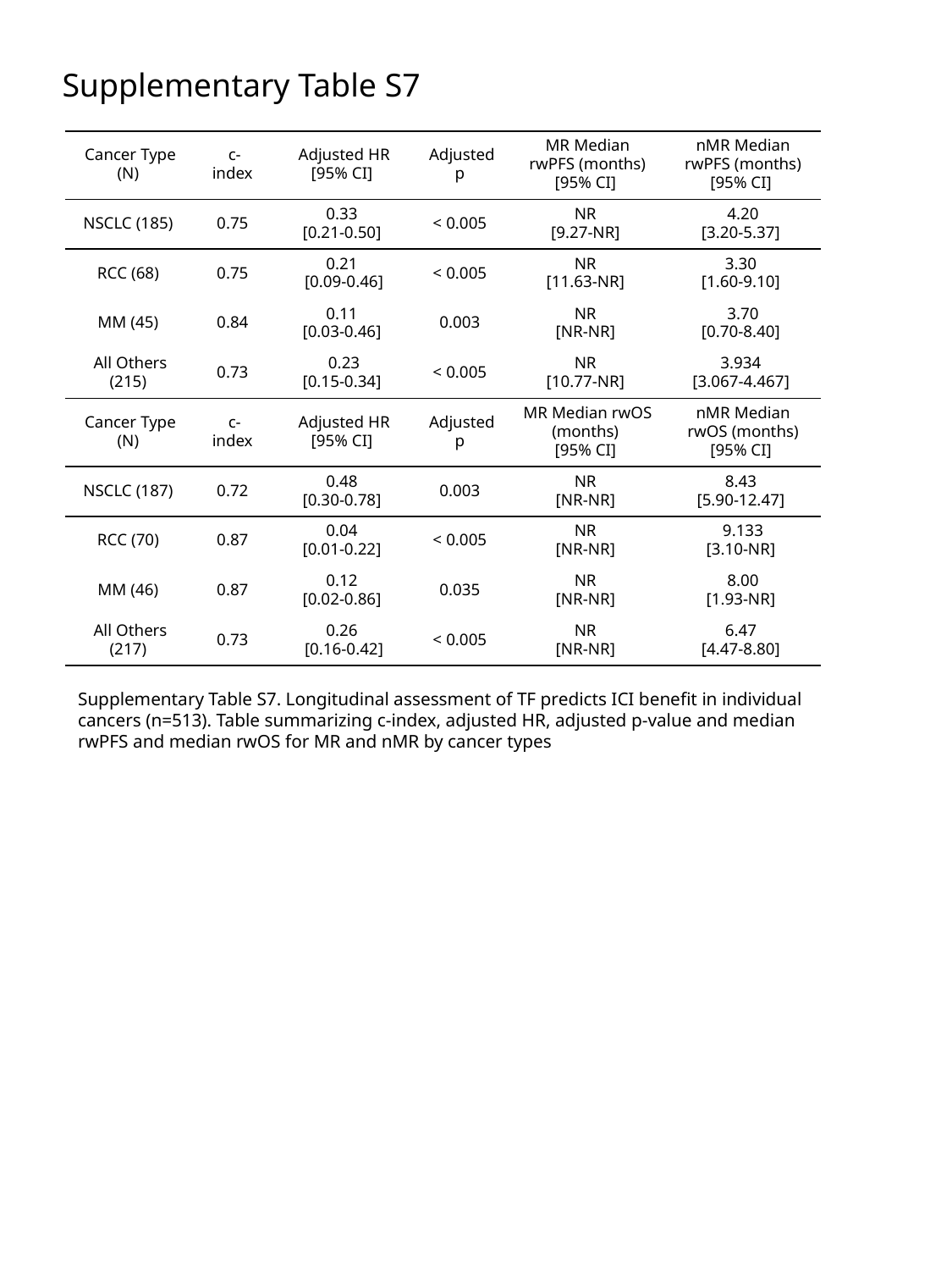

Supplementary Table S7
| Cancer Type (N) | c-index | Adjusted HR [95% CI] | Adjusted p | MR Median rwPFS (months) [95% CI] | nMR Median rwPFS (months) [95% CI] |
| --- | --- | --- | --- | --- | --- |
| NSCLC (185) | 0.75 | 0.33 [0.21-0.50] | < 0.005 | NR [9.27-NR] | 4.20 [3.20-5.37] |
| RCC (68) | 0.75 | 0.21 [0.09-0.46] | < 0.005 | NR [11.63-NR] | 3.30 [1.60-9.10] |
| MM (45) | 0.84 | 0.11 [0.03-0.46] | 0.003 | NR [NR-NR] | 3.70 [0.70-8.40] |
| All Others (215) | 0.73 | 0.23 [0.15-0.34] | < 0.005 | NR [10.77-NR] | 3.934 [3.067-4.467] |
| Cancer Type (N) | c-index | Adjusted HR [95% CI] | Adjusted p | MR Median rwOS (months) [95% CI] | nMR Median rwOS (months) [95% CI] |
| NSCLC (187) | 0.72 | 0.48 [0.30-0.78] | 0.003 | NR [NR-NR] | 8.43 [5.90-12.47] |
| RCC (70) | 0.87 | 0.04 [0.01-0.22] | < 0.005 | NR [NR-NR] | 9.133 [3.10-NR] |
| MM (46) | 0.87 | 0.12 [0.02-0.86] | 0.035 | NR [NR-NR] | 8.00 [1.93-NR] |
| All Others (217) | 0.73 | 0.26 [0.16-0.42] | < 0.005 | NR [NR-NR] | 6.47 [4.47-8.80] |
Supplementary Table S7. Longitudinal assessment of TF predicts ICI benefit in individual cancers (n=513). Table summarizing c-index, adjusted HR, adjusted p-value and median rwPFS and median rwOS for MR and nMR by cancer types
